# Meningococcal ACWYX conjugate vaccine in 2–29 year-olds in Mali and Gambia

**DOI:** 10.1056/NEJMoa2214924

**Published:** 2023-05-25

**Authors:** Fadima Cheick Haidara, Ama Umesi, Samba O. Sow, Magnus Ochoge, Fatoumata Diallo, Abdulazeez Imam, Youssouf Traore, Lucy Affleck, Moussa F. Doumbia, Bubacarr Daffeh, Mamoudou Kodio, Oghenebrume Wariri, Awa Traoré, Edrissa Jallow, Beate Kampmann, Dhananjay Kapse, Prasad S. Kulkarni, Asha Mallya, Sunil Goel, Pankaj Sharma, Annamraju D. Sarma, Nikhil Avalaskar, F. Marc LaForce, Mark R. Alderson, Abdi Naficy, Steve Lamola, Yuxiao Tang, Lionel Martellet, Nancy Hosken, Evangelos Simeonidis, Jo Anne Welsch, Milagritos D. Tapia, Ed Clarke

**Affiliations:** Centre pour le Developpement des Vaccins, Bamako, Mali; MRC Unit The Gambia at the London School of Hygiene and Tropical Medicine, Fajara, Banjul, The Gambia; Centre pour le Developpement des Vaccins, Bamako, Mali; MRC Unit The Gambia at the London School of Hygiene and Tropical Medicine, Fajara, Banjul, The Gambia; Centre pour le Developpement des Vaccins, Bamako, Mali; MRC Unit The Gambia at the London School of Hygiene and Tropical Medicine, Fajara, Banjul, The Gambia; Centre pour le Developpement des Vaccins, Bamako, Mali; MRC Unit The Gambia at the London School of Hygiene and Tropical Medicine, Fajara, Banjul, The Gambia; Centre pour le Developpement des Vaccins, Bamako, Mali; MRC Unit The Gambia at the London School of Hygiene and Tropical Medicine, Fajara, Banjul, The Gambia; Centre pour le Developpement des Vaccins, Bamako, Mali; MRC Unit The Gambia at the London School of Hygiene and Tropical Medicine, Fajara, Banjul, The Gambia; Centre pour le Developpement des Vaccins, Bamako, Mali; MRC Unit The Gambia at the London School of Hygiene and Tropical Medicine, Fajara, Banjul, The Gambia; MRC Unit The Gambia at the London School of Hygiene and Tropical Medicine, Fajara, Banjul, The Gambia; Serum Institute of India Pvt Ltd, Pune, India; Serum Institute of India Pvt Ltd, Pune, India; Serum Institute of India Pvt Ltd, Pune, India; Serum Institute of India Pvt Ltd, Pune, India; Serum Institute of India Pvt Ltd, Pune, India; Serum Institute of India Pvt Ltd, Pune, India; Serum Institute of India Pvt Ltd, Pune, India; Serum Institute of India Pvt Ltd, Pune, India; Center for Vaccine Innovation and Access, PATH, Seattle, WA 98121, USA; Center for Vaccine Innovation and Access, PATH, Seattle, WA 98121, USA; Center for Vaccine Innovation and Access, PATH, Seattle, WA 98121, USA; Center for Vaccine Innovation and Access, PATH, Seattle, WA 98121, USA; Center for Vaccine Innovation and Access, PATH, Seattle, WA 98121, USA; Center for Vaccine Innovation and Access, PATH, Seattle, WA 98121, USA; Center for Vaccine Innovation and Access, PATH, Seattle, WA 98121, USA; Center for Vaccine Innovation and Access, PATH, Seattle, WA 98121, USA; MRC Unit The Gambia at the London School of Hygiene and Tropical Medicine, PO Box 273, Fajara, Banjul, The Gambia

**Keywords:** bacterial meningitis, meningococcal conjugate vaccine, serogroup X, African meningitis belt

## Abstract

**Background:**

An effective, affordable, multivalent meningococcal conjugate vaccine is needed to prevent epidemic meningitis in the African meningitis belt. This trial assessed the safety and immunogenicity of NmCV-5, a pentavalent ACWYX vaccine.

**Methods:**

We conducted a phase 3, non-inferiority trial in healthy 2-29-year-olds in Mali and The Gambia. Participants were randomly assigned, 2:1, to receive a single intramuscular dose of NmCV-5 or MenACWY-D (Menactra). Immunogenicity was assessed at day 28. The non-inferiority of NmCV-5 compared to MenACWY-D was assessed based on differences in seroresponse rates (lower bound of 96% CI above −10%) or geometric mean titre (GMT) ratios (lower bound of 98.98% CI above 0.5). Serogroup X responses were compared to the lowest response to MenACWY-D serogroups.

**Results:**

1800 participants received NmCV-5 or MenACWY-D. NmCV-5 seroresponse rates ranged from 70.5% (95% CI 67.8–73.2) for serogroup A to 98.5% (95% CI 97.6–99.2) for serogroup W and serogroup X seroresponse rate was 97.2% (95% CI 96.0–98.1). The overall difference in seroresponse rates (NmCV-5–MenACYW-D) for the shared serogroups ranged from 1.2% (96%CI −0.3–3.1) for serogroup W to 20.5% (96%CI 15.4–25.6) for serogroup A. The overall GMT ratios (NmCV-5/MenACYW-D) for shared serogroups ranged from 1.7 (98.98%CI 1.5–1.9) for serogroup A to 2.8 (98.98%CI 2.3–3.5) for serogroup C. The serogroup X component of NmCV-5 generated seroresponses and GMTs that met the pre-specified non-inferiority criteria. Systemic adverse events were similar between groups (11.1% NmCV-5 and 9.2% MenACWY-D).

**Conclusions:**

NmCV-5 elicits comparable immune responses to all 4 serotypes in common with MenACWY-D as well as to serogroup X without evident safety concerns.

The Global Roadmap for Defeating Meningitis by 2030 was endorsed by the World Health Assembly in November 2020 and aims to eliminate epidemic bacterial meningitis, and to reduce rates of vaccine preventable disease by 50%, and death by 70%, before the end of the decade.^1^

In 2019, there were estimated to be over 2.5 million cases of meningitis worldwide, resulting in more than 236 000 deaths.^2^ The highest incidence and mortality rates for the disease occur across the African meningitis belt, stretching from The Gambia and Senegal in the west, to Ethiopia in the east, where meningitis epidemics, predominantly caused by *Neisseria meningitidis,* occur on a background of high endemic disease rates.^2,3^ While six serogroups of meningococcus (A, B, C, W, X, and Y) can cause invasive disease, serogroup A has historically been the most important cause of disease in this setting. However, following mass vaccination campaigns with MenAfriVac^®^, a conjugate vaccine developed to address this burden, through a partnership between the Serum Institute of India, WHO, and PATH, serogroup A disease has been virtually eliminated.^4–6^ Nonetheless, countries in the meningitis belt continue to record high rates of disease due to other serogroups. Large epidemics of meningitis caused by meningococcal serogroup C have occurred in Niger and north-western Nigeria and this serogroup continues to predominate in countries, including Chad, Mali, Burkina Faso and Togo, from which surveillance data are available.^6–11^ Epidemic serogroup W disease has also been reported including in Ghana and Togo, while serogroup X has additionally emerged having epidemic potential in the sub-region and elsewhere.^8,9,12–15^

Although four quadrivalent meningococcal ACWY conjugate vaccines are licensed and prequalified by WHO, their use in the African meningitis belt has been limited by supply and cost constraints.^14^ Furthermore, there are currently no licensed vaccines against meningococcal serogroup X.^13,14^ Thus, building upon the success of the Meningitis Vaccine Project which developed MenAfriVac, the Serum Institute of India and PATH have developed a pentavalent meningococcal ACWYX conjugate vaccine (NmCV-5), with the goal of eliminating meningococcal disease in sub-Saharan Africa. Supportive data on the safety and immunogenicity of NmCV-5 have been reported following a phase 1 trial in 18-45-year-olds in the United States, and a phase 2 trial in 12-16-month-olds in Mali.^16,17^

Here we report the results of a phase 3 trial of NmCV-5 conducted in 2- to 29-year-olds, the target age-groups for meningococcal outbreak response campaigns, in Mali and The Gambia. The trial aimed to demonstrate the safety and immunological non-inferiority of NmCV-5 compared to a licensed, WHO prequalified quadrivalent meningococcal conjugate vaccine (MenACWY-D [Menactra^®^, Sanofi Pasteur]). It provides data required for the licensure and WHO prequalification of the vaccine for future epidemic control.

## Methods

### Study design and participants

We conducted a two-center, phase 3, observer-blind, randomized, active-controlled, non-inferiority trial in Mali and The Gambia. Following screening for eligibility based on defined inclusion and exclusion criteria (Supplementary Material), 600 participants were enrolled in each of three age groups: 2-10-years, 11-17-years, 18-29-years. All participants (≥18 years), or their parents/guardians, provided written informed consent. Participants aged ≥13 years (Mali) or ≥12 years (The Gambia) also provided written assent. For full details of study conduct see protocol at nejm.org.

The responsibilities of the given authors for the design, conduct, analysis, and publication of the study are outlined in the Supplementary Material.

### Oversight

The trial was conducted in accordance with the Declaration of Helsinki and Good Clinical Practice Guidelines. It was approved by the research ethics committee of the Faculté de Médecine, de Pharmacie et d’Odonto-Stomatologie, Mali; the Institutional Review Board of the University of Maryland School of Medicine, USA; the Gambia Government/MRC Joint Ethics Committee, The Gambia; and the Western Institutional Review Board. Regulatory approval was obtained from Directorate of Pharmacy and Medicine, Mali and the Medicines Control Agency, The Gambia. A data safety monitoring board oversaw the trial.

### Randomization and blinding

Eligible participants within each age category were randomly assigned in a 2:1 ratio to receive either NmCV-5 (n=400) or MenACWY-D (n=200). Randomization was undertaken using a web-based system, according to a permuted block randomization scheme. Randomization, vaccine preparation and administration were undertaken by unblind personnel who were not involved in other participant-related procedures or endpoint collection. Parents, participants, and all other trial staff were blind to treatment allocation.

### Vaccines

A single 0.5 mL dose of NmCV-5 contains 5 µg of meningococcal serogroups A and X polysaccharides conjugated to tetanus toxoid and 5 µg of meningococcal serogroups C, W, and Y polysaccharides conjugated to recombinant cross-reactive material-197. A single dose of MenACWY-D contains 4 µg each of meningococcal A, C, W, and Y polysaccharides conjugated to diphtheria toxoid (Supplementary Material). The vaccines were administered by intramuscular injection into the deltoid muscle using a 23G 25mm needle.

### Objectives and endpoints

The trial had two primary objectives. First, to demonstrate the immune responses to meningococcal serogroups A, C, W and Y generated by NmCV-5 were non-inferior to those generated by MenACWY-D. Second, to demonstrate the immune responses to meningococcal serogroup X generated by NmCV-5 were non-inferior to the lowest immune response generated by MenACWY-D against serogroups A, C, W and Y. Comparison to the lowest response generated against the serogroups in MenACWY-D was made following regulatory agreement in the absence of a licensed serogroup X comparator vaccine. Serum samples collected pre- and on day 28 post-vaccination were tested for serogroup-specific serum bactericidal activity using rabbit complement (rSBA).^18,19^ Immune responses were defined in terms of two primary endpoints; serogroup-specific rSBA seroresponse rates and geometric mean titres (GMT) measured 28 days after vaccination. Seroresponse rate was defined as the percentage of participants with post-vaccination rSBA titre of ≥ 32 in those with a pre-vaccination titre of < 8 or at least four times as high as pre-vaccination titre in those with a pre-vaccination titre of ≥ 8. Secondary endpoints included the percentage of participants with rSBA titres ≥ 8 and ≥ 128 pre- and on day 28 post-vaccination, and data related to the safety and tolerability of NmCV-5. Details of the visit schedule are provided in the Supplementary Material.

Solicited injection-site and systemic adverse events were collected and graded for severity on the day of vaccination and for a further six days post-vaccination through home-visits conducted by trained fieldworkers. Unsolicited adverse events were collected by study clinicians from the day of vaccination and for a further 28 days post-vaccination and were graded for severity. Solicited systemic events and unsolicited events were judged by the investigator for relatedness to vaccination. Serious adverse events (SAE) were collected for 168 days post-vaccination (Supplementary Material).

### Statistical analysis

The immunological non-inferiority of NmCV-5 compared to MenACWY-D was assessed based on achieving the criteria set for either of the two primary endpoints. A prospective alpha allocation scheme was employed for multiplicity adjustment. One-sided significance levels of 0.02 and 0.0051 were applied to non-inferiority testing for seroresponse rates with a margin of −10% and GMTs with a margin of 0.5, respectively. The difference in the percentages of participants with serogroup-specific seroresponse between NmCV-5 and MenACWY-D (seroresponse_NmCV-5_ – seroresponse_MenACWY-D_) was calculated with its two-sided 96% confidence interval (CI) obtained using the Miettinen and Nurminen method.^20^ The ratio of the GMTs between the NmCV-5 and MenACWY-D (GMT_NmCV-5_/GMT_MenACWY-D_) against each serogroup were calculated with its two-sided 98.98% CI. For each serogroup, the log2-transformed rSBA titres were used to construct a two-sided 98.98% CI for the mean difference between the two vaccine groups using analysis of covariance with log2-transformed baseline titres as a covariate. Age, sex, and study site were evaluated for inclusion in the model using stepwise selection. The mean difference and corresponding 98.98% CI limits were exponentiated to obtain the ratio of GMTs and the corresponding 98.98% CI.

The sample size and power calculations are provided in the Supplementary Material. The primary immunogenicity and safety analyses were conducted on the per protocol and safety populations, respectively (Supplementary Material). All statistical analyses were performed using SAS^®^ software version 9.4.

## Results

### Trial Population

The first participants were recruited in August 2019. Safety follow-up to 168 days post-vaccination was completed in June 2021. Consent was provided for 1869 participants, of whom 1800 were eligible and were randomized and vaccinated (Supplementary Figure S1). Overall, 50.7% of participants were female, all were African, and 43.4% belonged to the Mandinka/Malinke ethnic group (Table 1). There were no notable differences in demographic or anthropometric parameters between vaccine groups in any age category. The participants in the study are considered representative of the target population for NmCV-5 (Supplementary Material)

### Immunogenicity results

The overall serogroup-specific seroresponse rates for serogroups ACWY 28 days following vaccination with NmCV-5 ranged from 70.5% (95% CI 67.8–73.2) for serogroup A to 98.5% (95% CI 97.6–99.2) for serogroup W (Table 2A). The serogroup X seroresponse rate was 97.2% (95% CI 96.0–98.1). The serogroup-specific seroresponse rate following vaccination with MenACWY-D, for the four included serogroups, ranged from 50.0% (95% CI 45.8–54.2) for serogroup A to 97.4% (95% CI 95.6–98.6) for serogroup W.

**Figure 1:**
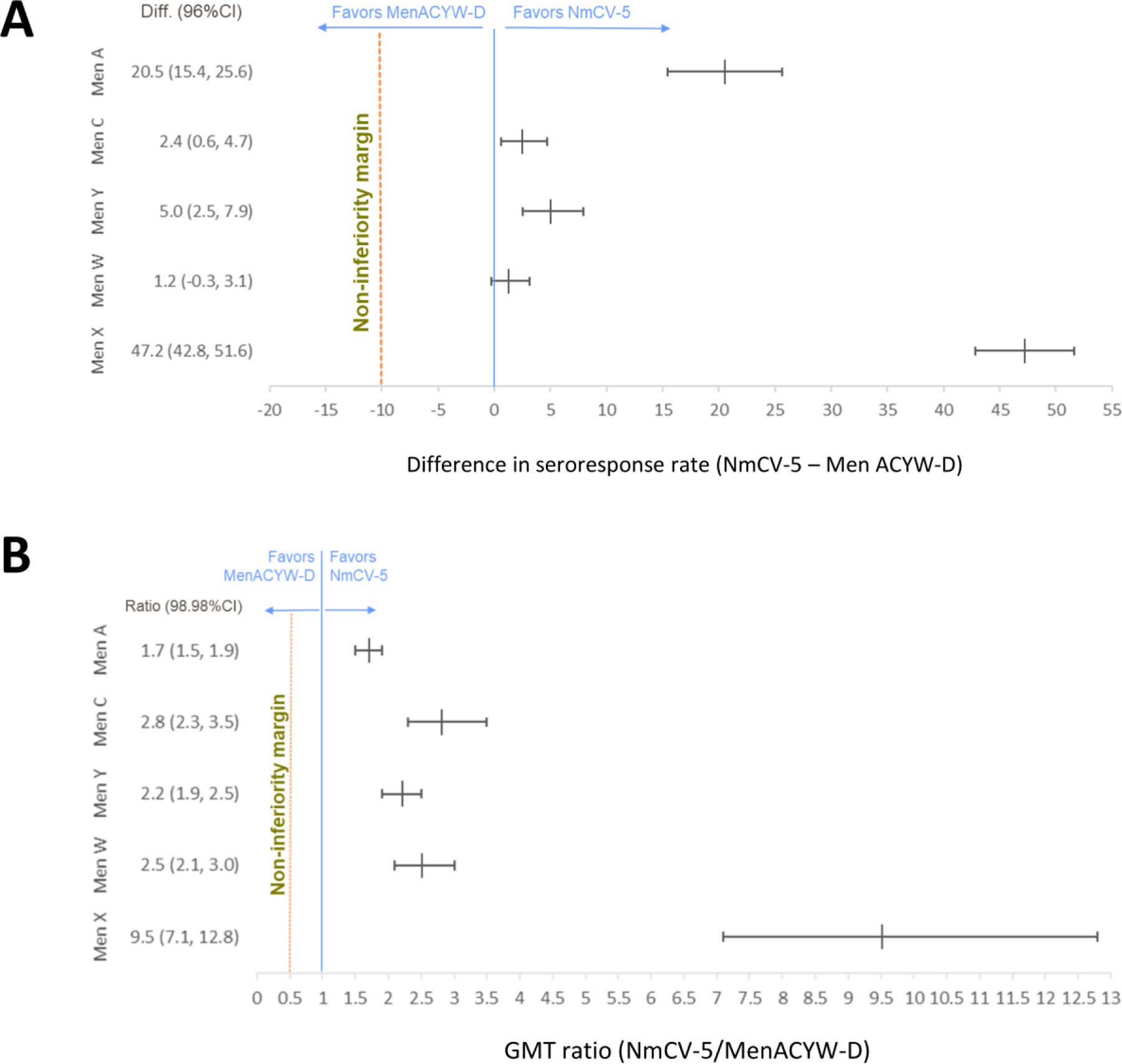
Overall non-inferiority analysis 28 days post-vaccination for seroresponse rates (A) and geometric mean titres (B). NmCV-5 – Serum Institute of India Pvt. Ltd pentavalent ACWYX meningococcal conjugate vaccine; MenACWY-D – Sanofi Pasteur quadrivalent meningococcal conjugate vaccine (Menactra^®^); For serogroup X the comparisons are between the seroresponse, or GMT generated against this serogroup by NmCV-5 and the lowest seroresponse or GMT generated by Men ACWY-D against the four serogroups it contains (serogroup A for seroresponse rates, serogroup C for GMT); per-protocol population

As the lowest seroresponse rate following MenACWY-D was to serogroup A, this was used as the comparator, for the purposes of the non-inferiority analysis, for serogroup X in NmCV-5. The difference in seroresponse rates for the shared serogroups ranged from 1.2% (96% CI −0.3–3.1) for serogroup W to 20.5% (96% CI 15.4–25.6) for serogroup A. The difference in the seroresponse rate comparing serogroup X in NmCV-5 to serogroup A in MenACWY-D was 47.2% (96% CI 42.8–51.6). The lower limit of the 96% CI was above the −10% non-inferiority margin for all serogroups for the overall population (Figure 1A) and in each age group. Thus, non-inferiority of NmCV-5 compared to MenACWY-D was demonstrated based on seroresponse rates.

The overall serogroup-specific rSBA GMT 28 days following vaccination with NmCV-5 ranged from 5587.2 (95% CI 5123.7–6092.5) for serogroup C to 31290.4 (95% CI 29222.2–33505.1) for serogroup X (Table 2B). The serogroup-specific rSBA GMT at the same timepoint following MenACWY-D for the four included serogroups ranged from 1854.9 (95% CI 1619.6–2124.4) for serogroup C to 12294.6 (95% CI 10778.9–14023.4) for serogroup W.

As the lowest rSBA GMT following MenACWY-D was to serogroup C this was used as the comparator, for the purposes of the non-inferiority analysis, for serogroup X in NmCV-5. The adjusted rSBA GMT ratio for the shared serogroups ranged from 1.7 (98.98% CI 1.5–1.9) for serogroup A to 2.8 (98.98% CI 2.3–3.5) for serogroup C. The adjusted GMT ratio comparing serogroup X in NmCV-5 to serogroup C in MenACWY-D was 9.5 (98.98% CI 7.1–12.8). The lower limit of the 98.98% CI was above the 0.5 non-inferiority margin for all serogroups in the overall population (Figure 1B) and in each age group. Thus, non-inferiority of NmCV-5 compared to MenACWY-D was demonstrated based on rSBA GMTs. Hence, the primary immunogenicity objective of the trial, to demonstrate the non-inferiority of NmCV-5 compared to MenACWY-D, was achieved in each age group based on both seroresponse rates and GMTs.

**Figure 2:**
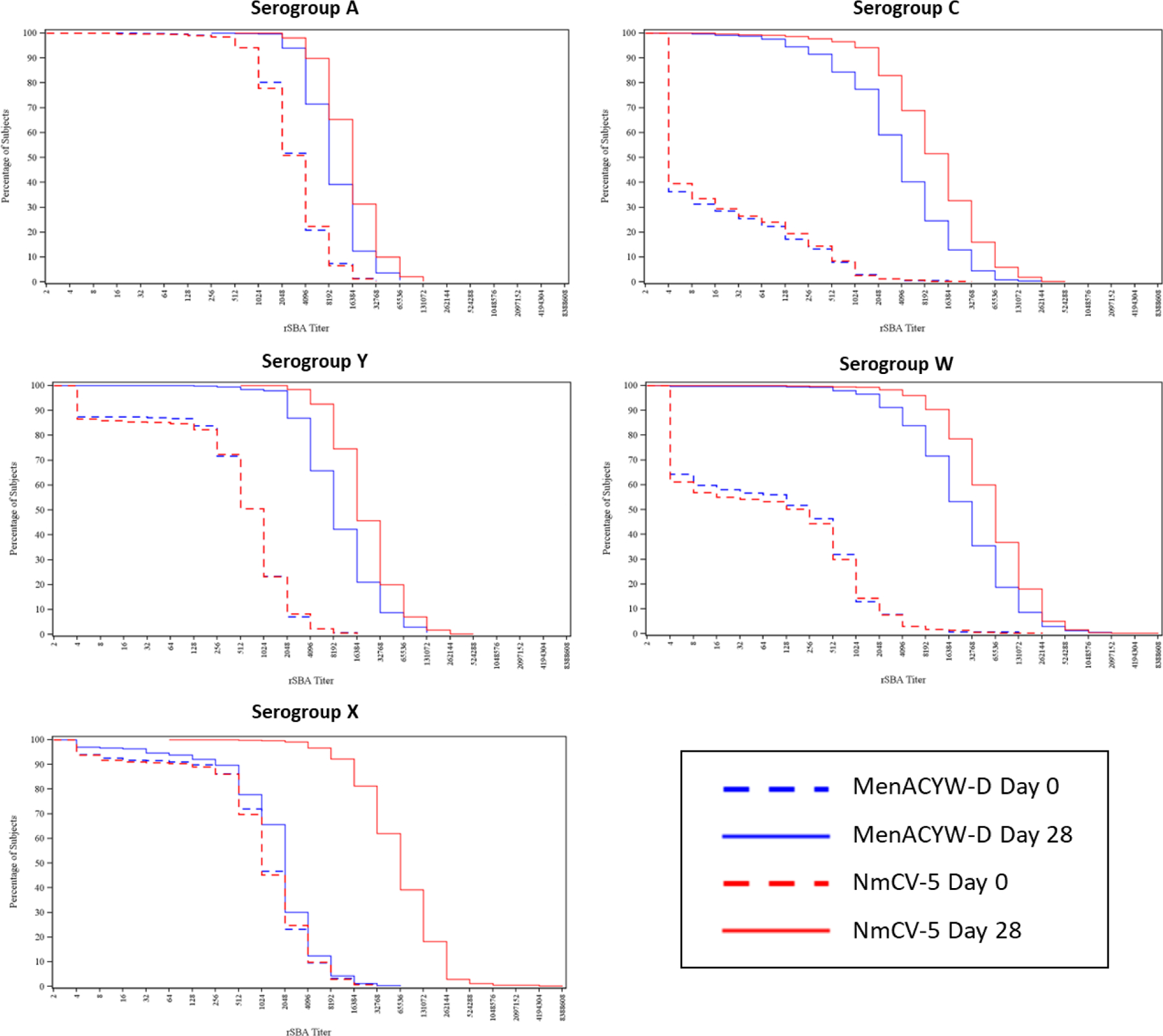
Reverse cumulative distributions curves pre- (day 0) and post-vaccination (day 28) Nm-CV-5 – Serum Institute of India Pvt. Ltd pentavalent ACWYX meningococcal conjugate vaccine; MenACWY-D – Sanofi Pasteur quadrivalent meningococcal conjugate vaccine (Menactra^®^).

The percentage of participants with baseline and post-vaccination serogroup-specific rSBA titres of ≥ 8 and ≥ 128 are provided in Supplementary Table S1. The geometric mean fold rises following NmCV-5 tended to be higher than those generated by MenACWY-D for all serogroups and in all age groups (Supplementary Table S2). While there were no notable differences in the distribution of rSBA titres at baseline, the proportion of participants above any given titre tended to be higher following NmCV-5 vaccination (Figure 2).

### Safety results

Overall, 312 (26.0%) of participants in the NmCV-5 group and 115 (19.2%) of participants in the MenACWY-D group (p=0.001) experienced at least one solicited injection site reaction (Table 3). Pain was most common, occurring in 311 (25.9%) and 115 (19.2%) of participants, respectively. Overall, 133 (11.1%) of participants in the NmCV-5 group and 55 (9.2%) of participants in the MenACWY-D group experienced a solicited systemic adverse event. All solicited events were mild or moderate in severity and resolved with no more than simple analgesia. Following vaccination with NmCV-5, 189 participants (15.8%) had a mild or moderate unsolicited adverse event compared to 99 (16.5%) of participants following vaccination with MenACWY-D. None of the unsolicited events were judged related to vaccination. The most common unsolicited events were upper respiratory tract infections, malaria, and pharyngitis, which occurred in 4.6%, 1.3% and 0.8% of participants, respectively (Supplementary Table S3). There were three serious adverse events following each vaccine, none of which were deemed by the investigator to be vaccine related. One 18-year-old participant in the MenACWY-D group died following trauma unrelated to the trial. Thirteen pregnancies were reported during study follow-up. Eleven women had normal deliveries without congenital anomalies. Two women chose to terminate their pregnancies.

## Discussion

This phase 3 trial demonstrated the immunological non-inferiority of NmCV-5 compared to the licensed, WHO pre-qualified quadrivalent meningococcal conjugate vaccine, MenACWY-D. Non-inferiority was demonstrated in all three age-groups based on both seroresponse rates and GMTs. The vaccine had a comparable safety profile to the licensed vaccine. These data are expected to support the licensure and WHO pre-qualification of NmCV-5, as a pentavalent meningococcal conjugate vaccine, including for serogroup X.

The licensure of meningococcal conjugate vaccines, including those targeting novel serogroups, based on immunogenicity rather than efficacy endpoints, is a well-established approach.^21,22^ Serum bactericidal antibodies, measured using human complement, were originally defined as a correlate of protection against invasive serogroup C disease in US military recruits.^23,24^ However, the standardized assay using rabbit complement was subsequently used to support the licensure and introduction of serogroup C conjugate vaccines in the UK.^21,25–27^ A short term, one-dose efficacy of 97% in teenagers and 92% in toddlers support the validity of this approach in the UK, while a rSBA titre of ≥ 8 or a four-fold rise in titres were identified as markers of vaccine-induced protection against this serogroup.^26,28^ In the UK and elsewhere, effectiveness of between 91% and 96% within 12 months of vaccination has been demonstrated in all age groups, with protection being sustained more consistently in those vaccinated beyond infancy.^29,30^

A comparable approach was used for the licensure of the meningococcal serogroup A conjugate vaccine, MenAfriVac.^21,22,31^ In the absence of a defined correlate of protection, and in the context of high baseline antibody titres, the requirement for a four-fold rise in rSBA was used as the primary endpoint.^31^ Based on enhanced surveillance leading up to and following the roll out of the vaccine across the meningitis belt, meningitis incidence has substantially decreased and serogroup A disease has all but disappeared. Burkina Faso recorded a 71% reduction in meningitis and a 99.8% reduction in serogroup A meningitis in the year following the MenAfriVac campaign.^32^ A 94% difference in the incidence of meningitis was also recorded in Chad within 4 to 6-months of vaccination.^33^ A study conducted across nine countries in the meningitis belt reported a 57% reduction in suspected meningitis and a more than 99% decrease in serogroup A meningitis associated with mass campaigns.^4^ Serogroup C and A conjugate vaccines also generate herd protection, indicating an impact on nasopharyngeal carriage as well as invasive disease.^34–37^

Finally, there are now early data on the effectiveness of MenACWY-D and other quadrivalent vaccines, also licensed based on immunogenicity. Analyzing serogroup C and serogroup Y breakthrough cases following the introduction of a single adolescent MenACWY-D vaccine dose in the US, vaccine effectiveness was estimated to be between 80 and 85%.^38^ A case-control study conducted in the same setting estimated a vaccine effectiveness of 79% within one year, and of 69% between one and three years following vaccination. The effectiveness against serogroup C was 79%, and against serogroup Y was 51% up to eight-years following vaccination.^39^ Following the introduction of an adolescent quadrivalent vaccine programme, predominantly using a tetanus toxoid conjugate (Nimenrix®, Pfizer) in the UK, an overall vaccine effectiveness of 94% has recently been reported, including effectiveness of 94% and 82% against serogroups W and Y respectively.^40^ The programme has also been shown to reduce pharyngeal carriage of meningococcus and is expected to generate herd protection.^41^ Thus, strong post-implementation data support the licensure of meningococcal conjugate vaccines based on immunogenicity rather than efficacy endpoints. The availability of such effectiveness data and the extensive use of MenACWY-D, as the first quadrivalent conjugate vaccine licensed, including as part of an outbreak response in West Africa, support the choice of the vaccine as the comparator in this study. Some differences in the immunogenicity of the four currently licensed quadrivalent vaccines have been reported. However, there are no data to suggest these translate into difference in effectiveness.^42^ The generally higher immune responses to NmCV-5 over the licensed comparator provides further reassurance with this regard.

The trial had several strengths. Both Mali and The Gambia are in the meningitis belt and are thus representative of a key future target population for NmCV-5, while the findings are also likely to translate to other settings. The consistency of the immune responses across age groups is also reassuring considering the future impact of the vaccine. Finally, the technology used in NmCV-5 production is based on cost-effective methods for carrier protein production, polysaccharide fermentation and purification, and chemical conjugation. Thus, the vaccine is expected to be made available at a cost lower than that of the existing quadrivalent vaccines.

The limitation of product licensure based on immunogenicity is acknowledged and generating effectiveness data for NmCV-5, particularly against serogroup X disease, will be important. Furthermore, data on the persistence of immune responses at six- and 12-months will be available in due course and important, particularly considering future routine use of NmCV-5 outside the epidemic response.

In addition, high baseline serogroup A GMTs, reflecting prior MenAfriVac campaigns and routine immunization programmes in Mali and The Gambia, limited the seroresponse rates to this serogroup. Nonetheless, post-vaccination titres were considerably above those demonstrated to provide protection against this serogroup, and responses of above 95% to the serogroup have previously been demonstrated in naïve toddlers.^16^

Based on the data from this trial, NmCV-5 may emerge as a tool to support meningococcal disease control, particularly across the meningitis belt of sub-Saharan Africa, and thus may contribute to epidemic elimination and the other goals of the Global Roadmap for Defeating Meningitis by 2030.

## Supplementary Material

Supplement

## Figures and Tables

**Table 1:** Demographic and anthropometric characteristics, safety population

	2−10 years			11−17 years			18−29 years			Overall		
	NmCV-5	MenACWY-D	Total	NmCV-5	MenACWY-D	Total	NmCV-5	MenACWY-D	Total	NmCV-5	MenACWY-D	Total
**Number analysed**	400	200	600	400	200	600	400	200	600	1200	600	1800
**Age (years):** Median (range)	6 (2 to 10)	5 (2 to 10)	6 (2 to 10)	13 (11 to 17)	13 (11 to 17)	13 (11 to 17)	22 (18 to 29)	21 (18 to 29)	21 (18 to 29)	13 (2 to 29)	13 (2 to 29)	13 (2 to 29)
**Sex n (%):** Female	179 (44.8)	94 (47.0)	273 (45.5)	187 (46.8)	102 (51.0)	289 (48.2)	240 (60.0)	111 (55.5)	351 (58.5)	606 (50.5)	307 (51.2)	913 (50.7)
**Race n (%): African**	400 (100.0)	200 (100.0)	600 (100.0)	400 (100.0)	200 (100.0)	600 (100.0)	400 (100.0)	200 (100.0)	600 (100.0)	1200 (100.0)	600 (100.0)	1800 (100.0)
**Ethnicity n (%)**												
Mandinka/Malinke	174 (43.5)	87 (43.5)	261 (43.5)	167 (41.8)	87 (43.5)	254 (42.3)	182 (45.5)	84 (42.0)	266 (44.3)	523 (43.6)	258 (43.0)	781 (43.4)
Bambara	92 (23.0)	41 (20.5)	133 (22.2)	84 (21.0)	42 (21.0)	126 (21.0)	58 (14.5)	40 (20.0)	98 (16.3)	234 (19.5)	123 (20.5)	357 (19.8)
Fula/Peulh	55 (13.8)	30 (15.0)	85 (14.2)	52 (13.0)	26 (13.0)	78 (13.0)	44 (11.0)	22 (11.0)	66 (11.0)	151 (12.6)	78 (13.0)	229 (12.7)
Other	79 (19.8)	42 (21.0)	121 (20.2)	97 (24.3)	45 (22.5)	142 (23.7)	116 (29.0)	54 (27.0)	170 (28.3)	292 (24.3)	141 (23.5)	433 (24.1)
**Anthropometry**												
Height (cm): mean (SD)	113.5 (17.3)	111.3 (16.4)	112.8 (17.1)	154.9 (11.6)	155.0 (11.5)	155.0 (11.5)	167.4 (8.9)	166.9 (8.7)	167.2 (8.8)	145.3 (26.5)	144.4 (27.0)	145.0 (26.7)
Weight (kg): mean (SD)	19.4 (6.8)	18.5 (5.8)	19.1 (6.5)	44.4 (12.8)	44.2 (12.1)	44.3 (12.5)	61.8 (12.0)	61.7 (12.5)	61.8 (12.1)	41.9 (20.5)	41.5 (20.6)	41.7 (20.5)

Nm-CV-5 – Serum Institute of India Pvt. Ltd pentavalent ACWYX meningococcal conjugate vaccine; MenACWY-D – Sanofi Pasteur quadrivalent meningococcal conjugate vaccine (Menactra^®^); n – number in respective category; cm – centimeters; kg – kilograms; SD – standard deviation

**Table 2:** Non-inferiority analysis based on (A) rSBA antibody seroresponse rates and (B) geometric mean titres, per protocol population

(A) Seroresponse rates												
	2 to 10-year-olds			11- to 17-year-olds			18- to 29-year-olds			Overall		
Serogroup	NmCV-5 n/N % (95% CI)	MenACWY-D n/N % (95% CI)	Difference^‡^ % (96% CI)	NmCV-5 n/N % (95% CI)	MenACWY-D n/N % (95% CI)	Difference^‡^ % (96% CI)	NmCV-5 n/N % (95% CI)	MenACWY-D n/N % (95% CI)	Difference^‡^ % (96% CI)	NmCV-5 n/N % (95% CI)	MenACWY-D n/N % (95% CI)	Difference^‡^ % (96% CI)
**A**	274/378	115/194		275/383	83/190		265/393	88/188		814/1154	286/572	
	72.5% (67.7–76.9)	59.3% (52.0–66.3)	13.2 (4.7–21.9)	71.8% (67.0–76.3)	43.7% (36.5–51.1)	28.1 (19.2–36.7)	67.4% (62.6–72.0)	46.8% (39.5–54.2)	20.6 (11.6–29.4)	70.5% (67.8–73.2)	50.0% (45.8–54.2)	20.5 (15.4–25.6)
**C**	379/385	183/189		368/371	177/182		362/377	171/185		1109/1133	531/556	
	98.4% (96.6–99.4)	96.8% (93.2–98.8)	1.6 (−1.0–5.5)	99.2% (97.7–99.8)	97.3% (93.7–99.1)	1.9 (−0.3–5.8)	96.0% (93.5–97.8)	92.4% (87.6–95.8)	3.6 (−0.5–8.9)	97.9% (96.9–98.6)	95.5% (93.4–97.1)	2.4 (0.6–4.7)
**W**	368/371	178/179		354/356	170/174		359/370	172/181		1081/1097	520/534	
	99.2% (97.7–99.8)	99.4% (96.9–100.0)	−0.2 (−2.0–2.5)	99.4% (98.0–99.9)	97.7% (94.2–99.4)	1.7 (−0.3–5.5)	97.0% (94.7–98.5)	95.0% (90.8–97.7)	2.0 (−1.4–6.7)	98.5% (97.6–99.2)	97.4% (95.6–98.6)	1.2 (−0.3–3.1)
**Y**	349/355	173/186		342/352	155/174		328/344	166/177		1019/1051	494/537	
	98.3% (96.4–99.4)	93.0% (88.3–96.2)	5.3 (1.8–10.3)	97.2% (94.8–98.6)	89.1% (83.5–93.3)	8.1 (3.5–14.1)	95.3% (92.6–97.3)	93.8% (89.2–96.9)	1.6 (−2.5–6.8)	97.0% (95.7–97.9)	92.0% (89.4–94.1)	5.0 (2.5–7.9)
**X** ^§^	376/386	19/182		364/371	14/177		359/374	15/148		1099/1131	48/507	
	97.4% (95.3–98.8)	10.4% (6.4–15.8)	38.1^§^ (30.9–45.7)	98.1% (96.2–99.2)	7.9% (4.4–12.9)	54.4^§^ (46.8–61.7)	96.0% (93.5–97.7)	10.1% (5.8–16.2)	49.2^§^ (41.4–56.8)	97.2% (96.0–98.1)	9.5% (7.1–12.4)	47.2^§^ (42.8–51.6)
**(B) Geometric mean titre**												
	**2 to 10-year-olds**			**11- to 17-year-olds**			**18- to 29-year-olds**			**Overall**		
**Serogroup**	**NmCV-5 n GMT (95% CI)**	**MenACWY-D n GMT (95% CI)**	**GMT ratio^¶^ (98.98% CI)**	**NmCV-5 n GMT (95% CI)**	**MenACWY-D n GMT (95% CI)**	**GMT ratio^¶^ (98.98% CI)**	**NmCV-5 n GMT (95% CI)**	**MenACWY-D n GMT (95% CI)**	**GMT ratio^¶^ (98.98% CI)**	**NmCV-5 n GMT (95% CI)**	**MenACWY-D n GMT (95% CI)**	**GMT ratio^¶^ (98.98% CI)**
**A**	388	199		389	192		395	192		1172	583	
	9250.1 (8529.3–100031.8)	5682.7 (5079.4–6357.6)	1.7 (1.4–2.0)	9463.9 (8762.4–10221.7)	4871.0 (4349.1–5455.6)	2.0 (1.7–2.4)	5900.3 (5419.2–6424.2)	3796.9 (3363.2–4286.7)	1.5 (1.2–1.8)	8009.9 (7631.7–8407.0)	4729.7 (4420.0–5061.2)	1.7 (1.5–1.9)
**C**	396	197		396	198		398	193		1190	588	
	3084.7 (2684.4–3544.7)	1020.4 (814.6–1278.3)	2.7 (2.0–3.7)	8021.7 (6973.0–9228.2)	2906.5 (2322.2–3637.8)	2.8 (2.0–3.9)	7040.2 (6036.8–8210.5)	2153.6 (1703.7–2722.4)	3.1 (2.2–4.5)	5587.2 (5123.7–6092.5)	1854.9 (1619.6–2124.4)	2.8 (2.3–3.5)
**W**	396	199		395	197		394	193		1185	589	
	28888.0 (25399.7–32855.3)	11208.2 (8887.3–14135.2)	2.4 (1.8–3.4)	30280.0 (26538.4–34549.0)	13453.9 (10845.1–16690.2)	2.4 (1.7–3.4)	27773.5 (24082.4–32030.4)	12336.7 (9713.9–15667.8)	2.4 (1.7–3.4)	28963.4 (26804.6–31295.9)	12294.6 (10778.9–14023.4)	2.5 (2.1–3.0)
**Y**	398	198		397	199		391	194		1186	591	
	10768.1 (9831.2–11794.3)	4362.4 (3701.0–5142.0)	2.3 (1.9–2.9)	11256.2 (10286.9–12317.0)	5154.6 (4359.4–6095.0)	2.1 (1.7–2.7)	10518.3 (9450.9–11706.2)	4967.7 (4227.8–5837.0)	2.0 (1.6–2.6)	10844.8 (10260.2–11462.8)	4815.6 (4380.9–5293.4)	2.2 (1.9–2.5)
**X** ^†^	399	187		392	180		396	156		1187	523	
	39737.0 (35838.0–44060.2)	1031.6 (845.1–1259.3)	17.5^†^ (8.4–36.6)	44572.8 (39929.6–49756.0)	835.0 (682.6–1021.4)	9.9^†^ (6.3–15.6)	17327.5 (15369.3–19535.2)	426.7 (311.7–584.2)	4.9^†^ (3.3–7.2)	31290.4 (29222.2–33505.1)	737.1 (641.3–847.4)	9.5^†^ (7.1–12.8)

rSBA – rabbit complement serum bactericidal activity; NmCV-5 – Serum Institute of India Pvt. Ltd pentavalent ACWYX meningococcal conjugate vaccine; MenACWY-D – Sanofi Pasteur quadrivalent meningococcal conjugate vaccine (Menactra^®^); n/N – number of participants with a rSBA seroresponse between pre-vaccination and day 28 post-vaccination samples/number of evaluable participants; CI – confidence interval; GMT – geometric mean titre

‡ - NmCV-5 seroresponse rate minus MenACWY-D seroresponse rate

§ - difference between serogroup X seroresponse rate following NmCV-5 and lowest seroresponse rate to serogroups A, C, W and Y following MenACWY-D (i.e., serogroup A in all cases)

¶ - NmCV-5 GMT/MenACWY-D GMT

† - Ratio of serogroup X GMT following NmCV-5 and the lowest GMT to serogroups A, C, W and Y following MenACWY-D (i.e., serogroup C in all cases); Seroresponse was defined as a post-vaccination rSBA titre of ≥ 32 in participants with a pre-vaccination rSBA titre of < 8; or at least a 4-fold increase over baseline in the post-vaccination rSBA titre in participants who had a pre-vaccination rSBA titre of ≥ 8.; The 95% CIs around seroresponse rates for each treatment group were calculated by using the Clopper-Pearson method. The 2-sided 96% CIs for the difference (NmCV-5 - Menactra) in percentages between the 2 groups were constructed using the Miettinen and Nurminen method. Post-vaccination GMT and 95% CI were calculated by exponentiating the corresponding log_2_-transformed mean and its 2-sided 95% CI. The log_2_-transformed rSBA titres were used to construct a 2-sided 98.98% CI for the mean difference between the 2 vaccine groups using analysis of covariance (ANCOVA). The mean difference and corresponding 98.98% CI limits were exponentiated to obtain the GMT ratio (GMT_NmCV-5_/GMT_MenACWY-D_) and the corresponding 98.98% CI. ANCOVA included log_2_-transformed baseline titres, age, sex, and study site as a covariate. Interaction terms for treatment group and baseline titres, treatment group and age, treatment group and study site, baseline titres and age, baseline titres and study site were also included in the model.

**Table 3:** Participants reporting solicited and unsolicited adverse events, safety population

	2 to 10-year-olds		11- to 17-year-olds		18- to 29-year-olds		Overall	
	NmCV-5 N=400 n (%)	MenACWY-D N=200 n (%)	NmCV-5 N=400 n (%)	MenACWY-D N=200 n (%)	NmCV-5 N=400 n (%)	MenACWY-D N=200 n (%)	NmCV-5 N=1200 n (%)	MenACWY-D N=600 n (%)
**Solicited adverse events** ^1^								
**Injection site adverse events**								
**Any**^§^	80 (20.1)	19 (9.5)	114 (28.5)	45 (22.5)	118 (29.5)	51 (25.6)	312 (26.0)	115 (19.2)
Any grade ≥ 3	0 (0.0)	0 (0.0)	0 (0.0)	0 (0.0)	0 (0.0)	0 (0.0)	(0.0)	0 (0.0)
**Pain**^§^	79 (19.8)	19 (9.5)	114 (28.5)	45 (22.5)	118 (29.5)	51 (25.6)	311 (25.9)	115 (19.2)
**Swelling/induration**	3 (0.8)	1 (0.5)	0 (0.0)	0 (0.0)	1 (0.3)	1 (0.5)	4 (0.3)	2 (0.3)

**Systemic adverse event**								
**Any**^¶^	30 (7.5)	8 (4.0)	50 (12.5)	24 (12.0)	53 (13.3)	23 (11.6)	133 (11.1)	55 (9.2)
Any grade ≥ 3	0 (0.0)	0 (0.0)	0 (0.0)	0 (0.0)	0 (0.0)	0 (0.0)	0 (0.0)	0 (0.0)
**Systemic adverse events (<6 years)**	N = 185	N = 110						
Fever	2 (1.1)	1 (0.9)	-	-	-	-	2 (1.1)	1 (0.9)
Drowsiness	3 (1.6)	0 (0.0)	-	-	-	-	3 (1.6)	0 (0.0)
Irritability	4 (2.2)	1 (0.9)	-	-	-	-	4 (2.2)	1 (0.9)
Anorexia	2 (1.1)	2 (1.8)	-	-	-	-	2 (1.1)	2 (1.8)
Diarrhoea	2 (1.1)	1 (0.9)	-	-	-	-	2 (1.1)	1 (0.9)
**Systemic adverse events** (≥**6 years)**	N = 214	N = 90						
Fever	5 (2.3)	0 (0.0)	7 (1.8)	2 (1.0)	1 (0.3)	2 (1.0)	13 (1.3)	4 (0.8)
Fatigue	2 (0.9)	0 (0.0)	11 (2.8)	4 (2.0)	25 (6.3)	12 (6.0)	38 (3.7)	16 (3.3)
Headache	14 (6.5)	2 (2.2)	32 (8.0)	17 (8.5)	27 (6.8)	10 (5.0)	73 (7.2)	29 (5.9)
Myalgia	3 (1.4)	1 (1.1)	11 (2.8)	3 (1.5)	8 (2.0)	9 (4.5)	22 (2.2)	13 (2.7)
Arthralgia	2 (0.9)	0 (0.0)	5 (1.3)	1 (0.5)	6 (1.5)	2 (1.0)	13 (1.3)	3 (0.6)
Anorexia	1 (0.5)	1 (1.1)	5 (1.3)	1 (0.5)	7 (1.8)	5 (2.5)	13 (1.3)	7 (1.4)
Diarrhoea	1 (0.5)	0 (0.0)	4 (1.0)	1 (0.5)	3 (0.8)	0 (0.0)	8 (0.8)	1 (0.2)

**Unsolicited adverse event** ^2^								
**Any**	81 (20.3)	36 (18.0)	44 (11.0)	25 (12.5)	64 (16.0)	38 (19.0)	189 (15.8)	99 (16.5)
Any grade ≥ 3	0 (0.0)	0 (0.0)	0 (0.0)	0 (0.0)	0 (0.0)	0 (0.0)	0 (0.0)	0 (0.0)
**Serious adverse events**^3^	0 (0.0)	2 (1.0)	0 (0.0)	0 (0.0)	3 (0.8)	1 (0.5)	3 (0.3)	3 (0.5)
Vaccine-related serious adverse events	0 (0.0)	0 (0.0)	0 (0.0)	0 (0.0)	0 (0.0)	0 (0.0)	0 (0.0)	0 (0.0)

Nm-CV-5 – Serum Institute of India Pvt. Ltd pentavalent ACWYX meningococcal conjugate vaccine; MenACWY-D – Sanofi Pasteur quadrivalent meningococcal conjugate vaccine (Menactra^®^)

§ Overall injection site reactions and pain at the injection site were higher in the NmCV-5 than the MenACWY-D group (p = 0.001 in both cases [Fisher’s exact test])

¶1 Collected for seven days after vaccination; the denominator for the percentages was 399 for 2 to10-year-olds in the NmCV-5 group, 199 for 18 to 29-year-olds in the MenACWY-D group, 1199 for the overall NmCV-5 group, 599 for the overall MenACWY-D group, and respective N for other combinations of age group and vaccine group

2 Collected for 28 days after vaccination

3 Collected for 168 days after vaccination.
