# Supplementary material for: Meningococcal ACWYX conjugate vaccine in 2–29 year-olds in Mali and Gambia
Source: N Engl J Med. Author manuscript; Available in PMC 2023 Nov 6. (PMC10627475; doi:10.1056/NEJMoa2214924)
Supplement: Supplement [file NIHMS1874118-supplement-Supplement.pdf]

Supplementary Appendix: *Immunogenicity and safety of a meningococcal ACWYX conjugate vaccine in 2- to 29-year-olds in Mali and The Gambia*

Contents

List of authors **3**

Eligibility criteria **4**

Description of Study Population 4

Inclusion Criteria 4

Exclusion Criteria 5

Vaccines **7**

NmCV-5 7

Men ACWY-D 7

Visit Schedule **9**

Laboratory assays **10**

Safety data collection **11**

Solicited local and systemic adverse events 11

Table: Grading scale to assess the severity of solicited adverse events for participants less than six years of age 11

Table: Grading scale to assess the severity of solicited adverse events for participants six years of age or older 12

Unsolicited adverse events 13

Statistical analysis **14**

Sample size calculation 14

Immunogenicity 14

Safety 15

Analysis populations 16

Representativeness of Study Participants **17**

Supplementary figures **18**

Figure S1: Screening, enrolment, and follow-up of participants 18

Supplementary tables **19**

Immunogenicity 19

Table S1: Percentage of participants with an rSBA titre >= 8 and >= 128 on the day of vaccination and on day 28 post-vaccination, per protocol population 19

Table S2: Baseline and post-vaccination serogroup-specific rSBA GMT and geometric mean fold rise, per protocol population 20

Safety 22

Table S3: Unsolicited adverse events by MedDRA[1] preferred term recorded in at least five participants - safety population... 22

List of authors

1. Fadima Cheick Haidara, M.D.
2. Ama Umesi, M.B.B.S.
3. Samba O. Sow, M.D.
4. Fatoumata Diallo, M.D.
5. Youssouf Traore, M.D.
6. Moussa F. Doumbia, M.D.
7. Mamoudou Kodio, Pharm.D.
8. Awa Traoré, Pharm.D.,
9. Magnus Ochoge, M.B.B.Ch.
10. Abdulazeez Imam, M.B.B.S.
11. Lucy Affleck, M.B.Ch.B.
12. Bubacarr Daffeh, M.B.Ch.B.
13. Oghenebrume Wariri, M.B.B.S.
14. Edrissa Jallow, B.Sc.
15. Beate Kampmann, Ph.D.
16. Dhananjay Kapse, M.D.
17. Prasad S Kulkarni, M.D.
18. Asha Mallya, Ph.D.
19. Sunil Goel, Ph.D.
20. Pankaj Sharma, M.Sc.
21. Annamraju D. Sarma, Ph.D.
22. Nikhil Avalaskar, M.Pharm.
23. F. Marc LaForce, M.D.
24. Mark R. Alderson, Ph.D.
25. Abdi Naficy, M.D.
26. Steve Lamola, M.D.
27. Yuxiao Tang, Ph.D.
28. Lionel Martellet, M.A.
29. Nancy Hosken, Ph.D.
30. Evangelos Simeonidis, Ph.D.
31. Jo Anne Welsch, Ph.D.
32. Milagritos D. Tapia, M.D.
33. Ed Clarke, M.B.Ch.B.

The trial was designed by authors 3, 16, 17, 24, 25, 27, 32 and 33. The data were collected by authors 1, 2, 4-14, 32, and 33. Author 27 was responsible for the analysis. The last author (33) wrote the first draft of the manuscript. All authors reviewed and provided input into the manuscript. All authors had access to the data reported. All the authors approved the final draft of the manuscript and vouch for the completeness and accuracy of the data and analyses. The last author made the final decision to submit the manuscript for publication.

Eligibility criteria

Description of Study Population

A total of 1800 healthy males or non-pregnant females 2 to 29 years of age are to be accrued in Mali and The Gambia, with 600 subjects in each of three age groups (2 to 10 years, 11 to 17 years, and 18 to 29 years).

Inclusion Criteria

Each subject must meet all the following inclusion criteria:

1. Male or non-pregnant female 2 through 29 years of age, inclusive, at the time of study IP administration.
2. Signed or fingerprinted or personally marked written informed consent obtained from subjects at least 18 years of age or from their parent/guardian for subjects less than 18 years of age with additional subject assent obtained as appropriate for participating community (i.e. subjects at least 13 years of age in Mali or at least 12 years of age in The Gambia).
3. Subject or parent/guardian with subject reside in study site area and are able and willing to adhere to all protocol visits and procedures.
4. Female subjects of childbearing potential* must have practiced adequate contraception** for 28 days prior to study IP administration and agree to continue adequate contraception until completion of their Day 29 visit.

   * Females can be considered not of childbearing potential only if they are pre-menarche and less than 12 years of age, or with current bilateral tubal ligation or occlusion, or post-hysterectomy, or post-bilateral ovariectomy, or post-menopause.

   ** Adequate contraception is defined as a contraceptive method with failure rate of less than 1% per year when used consistently and correctly and when applicable, in accordance with the product label as follows:

   - Abstinence from penile-vaginal intercourse when this is the preferred and usual lifestyle
   - Combined estrogen and progesterone oral contraceptives
   - Injectable progestogen
   - Implants of etonogestrel or levonorgestrel
   - Contraceptive vaginal ring
   - Percutaneous contraceptive patches
   - Intrauterine device or intrauterine system

- Male partner (sole partner for subject) sterilized
- Male condom combined with a vaginal spermicide (foam, gel, film, cream or suppository), and/or progesterone alone oral contraceptive

Female subjects of childbearing potential must have a negative pregnancy test within 24 hours prior to study IP administration.

Exclusion Criteria

Each subject must not meet any one of the following exclusion criteria:

1. Acute illness, at the time of study IP administration (once acute illness is resolved, if appropriate, as per investigator assessment, subject may be re-revaluated for eligibility).
2. Recorded fever (for eligibility purpose defined as a body temperature greater than 37.5°C) within 3 days prior to study IP administration (once fever/acute illness is resolved, if appropriate, as per investigator assessment, subject may be re-revaluated for eligibility).
3. Previous immunization with a *Neisseria meningitidis* vaccine other than MenAfriVac® during the previous five years.
4. Current or previous, confirmed disease caused by *Neisseria meningitidis*.
5. Household contact with or intimate exposure to an individual with any laboratory confirmed *Neisseria meningitidis* infection within 90 days prior to study IP administration.
6. Known hypersensitivity to any component of the study IPs (i.e., NmCV-5 or Men ACWY-D).
7. History of significant hypersensitivity reactions to any previous vaccine.
8. Administration of any vaccine other than study IPs within 28 days prior to study IP administration or planned administration prior to completion of the study Day 29 visit.
9. Administration of any investigational drug within 30 days prior to study IP administration or planned administration during the study period.
10. Unwilling to avoid (or their child to avoid, if the subject) the ingestion of herbal or other traditional medications during the study period.
11. Administration of immunoglobulin or any blood product within 90 days prior to study IP administration or planned administration during the study period.
12. Administration of immunosuppressants or other immune-modifying agents within 90 days prior to study IP administration (e.g., systemic corticosteroids at doses equivalent to ≥ 0.5 mg/kg/day of prednisone for more than 14 days; topical steroids including inhaled and intranasal steroids are not exclusionary).

13. Administration of systemic antibiotic treatment within 3 days prior to study IP administration.
14. Any history of or evidence for chronic clinically significant (as per investigator assessment) disorder or disease (including, but not limited to, immunodeficiency, autoimmunity, malnutrition*, congenital abnormality, bleeding disorder, and pulmonary, cardiovascular, metabolic, neurologic, renal, or hepatic disease).

    *Other than the exclusionary clinical diagnosis of malnutrition for all subjects, in children 2 to 5 years of age, malnutrition is also defined as a weight-for-height Z-score of less than -3 as per WHO reference standards.
15. Any history of human immunodeficiency virus, chronic hepatitis B or chronic hepatitis C infections.
16. History of meningitis, seizures, Guillain-Barré syndrome (GBS), or other neurological disorders.
17. History of or family history of congenital or hereditary immunodeficiency.
18. Any condition that in the opinion of the investigator might compromise the safety or well-being of the subject or compromise adherence to protocol procedures or interfere with planned safety and immunogenicity assessments.
19. Pregnancy (in the absence of reproductive toxicity information, pregnant women cannot not be enrolled).
20. Previous inclusion in the study of five immediate family members (i.e., biological father, mother, subject, and brothers and sisters may be included up to a maximum of five members from the same immediate family).

Vaccines

NmCV-5

The NmCV-5 investigational vaccine, manufactured by the Serum Institute of India Private Limited, is a fixed-combination available as a lyophilized powder containing meningococcal serogroups A and X polysaccharides conjugated to TT and meningococcal serogroups C, W and Y polysaccharides conjugated to CRM_{197} protein. The vaccine will be reconstituted with normal saline just prior to administration. *N. meningitidis* A, C, W, Y, and X polysaccharides are produced by cultivating cells in fed batch fermentation and purified after separation. Tetanus toxin is derived from *Clostridium tetani* grown in a modified medium and purified. CRM is expressed by a *Pseudomonas fluorescens* strain and used after it is purified. Meningococcal polysaccharides are covalently conjugated to TT or CRM and purified to make the final formulated vaccine. The NmCV-5 lyophilized powder component contains sucrose, sodium citrate and trometamol as excipients. No preservative or adjuvant is present in the vaccine. This component is presented as a freeze-dried powder in a five-dose vial.

0.9% Sodium Chloride (normal saline) used to prepare NmCV-5 is a clear, colourless liquid. A volume of 3.1 mL will be used to reconstitute the lyophilized component of NmCV-5. After reconstitution, a single 0.5 mL dose of NmCV-5 injectable solution will have the following composition:

Composition of a single 0.5 mL dose of NmCV-5

Meningococcal A polysaccharide [1]	5 µg
Meningococcal C polysaccharide [2]	5 µg
Meningococcal W polysaccharide [2]	5 µg
Meningococcal Y polysaccharide [2]	5 µg
Meningococcal X polysaccharide [1]	5 µg
Sucrose	2.42 mg
Sodium Citrate	0.40 mg
TRIS (Trometamol)	0.098 mg
0.9% Sodium Chloride	*quantum satis*
Tetanus Toxoid	7.8 to 33.4 mcg
CRM_{197}	11.7 to 50.1 µg

[1] Each polysaccharide conjugated to TT; [2] Each polysaccharide conjugated to CRM_{197}

Men ACWY-D

The control vaccine is the licensed meningococcal serogroups ACWY conjugate vaccine (Menactra®), manufactured by Sanofi Pasteur Incorporated. Men ACWY-D is supplied as a single 0.5 mL dose formulated in sodium phosphate buffered isotonic sodium chloride solution to contain 4 µg each of

meningococcal A, C, W, and Y polysaccharides conjugated to approximately 48 µg of diphtheria toxoid protein carrier. No preservative or adjuvant is added during manufacture. Each 0.5 mL dose contains:

Composition of a single 0.5 mL dose of MenACWY-D

Meningococcal A polysaccharide	4 µg
Meningococcal C polysaccharide	4 µg
Meningococcal W polysaccharide	4 µg
Meningococcal Y polysaccharide	4 µg
Diphtheria toxoid protein total	48 µg
Sodium Phosphate	0.7 mg
Sodium Chloride	4.35 mg

Each 0.5 mL dose may also contain residual amounts of formaldehyde of less than 2.66 µg

Visit Schedule

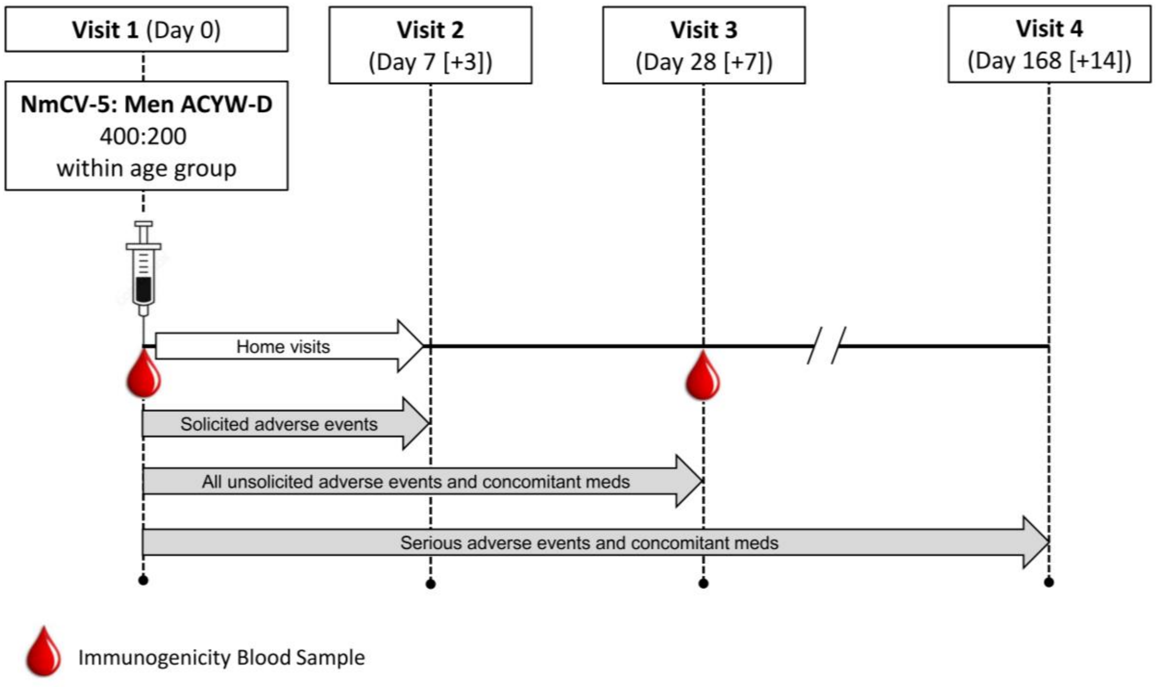

[ ] per-protocol window period

Laboratory assays

Clinical serum samples were tested by baby rabbit complement Serum Bactericidal Assay (rSBA) to assess the level of functional antibodies to meningococcal serogroups A, C, W, Y, and X (referred to as MenA, MenC, MenW, MenY and MenX, respectively). The *N. meningiditis* strains M99.243594 (MenA), L94.5016 (MenC), M01.24115 (MenW), M03.24115 (MenY) and BF 2/97 (MenX) were obtained from Public Health England Laboratory, Manchester, UK.

All clinical serum samples were tested following a validated rSBA method for each of the serogroups at Nexelis, 525 Boulevard Cartier Ouest, Laval, QC, Canada H7V 3S8, Canada. Serum samples were heat inactivated at 56°C for 30 minutes to inactivate endogenous complement components. Serial dilutions of the serum samples were prepared in a 96-well plate. A defined concentration of *N. meningiditis* bacteria and active baby rabbit complement were added sequentially to the sera dilutions and incubated at 37 ± 2°C for 60 ± 5 minutes. An agar overlay medium was then added to the wells followed by an incubation overnight at 37 ± 2° C, depending on the strain being used. The following day, images from each well were captured and the number of colony forming units (CFUs) were determined by an automated system. The number of resulting bacterial colonies present in the wells is inversely proportional to the level of functional antibodies present in the serum, which is directly proportional to the immunological response of the subject.

Two positive Quality Control sera were tested on each plate to monitor the assay stability. The results were monitored on a QC chart (Levey-Jennings) to ensure the validity criteria of the assay, as per Nexelis' standard procedures.

Baby rabbit complement lots were obtained from Pel-Freez Biologicals, Arkansas, US.

The rSBA method has a lower limit of quantitation (LLOQ) of 1:4.

Acceptance criteria, as described within Nexelis' standard assay procedures, were applied during sample testing to evaluate run and sample validity.

Assays were executed by qualified analysts, using appropriately validated equipment, as per Nexelis' standard procedures.

Assay stability (proficiency panel and Levey-Jennings Quality Control (QC) charts) was monitored over time as per Nexelis' standard procedures. Proficiency panels were tested at predefined timepoints for all 5 serogroups (A, C, W, Y & X) and all serogroups met the acceptance criteria as per the assay stability monitoring plan.

All clinical trial serological activities were audited by Nexelis Quality Assurance (QA) department before release to the sponsor. A PATH subcontracted independent audit of Nexelis was performed after clinical testing. Based on the audits executed, this project was performed in accordance with applicable

International Council for Harmonization (ICH) Good Clinical Practice (GCP) regulatory requirements and Research Quality Association (RQA) Good Clinical Laboratory Practice (GCLP) guidelines.

Safety data collection

Solicited local and systemic adverse events

Table: Grading scale to assess the severity of solicited adverse events for participants less than six years of age

Solicited AE	Grade	Definition
Pain at injection site	1	Pain or tenderness causing no or minimal limitation of use of limb
	2	Pain or tenderness causing greater than minimal limitation of use of limb
	3	Pain or tenderness causing inability to perform usual social & functional activities
	4	Pain or tenderness causing inability to perform basic self-care function or hospitalization indicated
Swelling/ induration at injection site	1	≤ 25 mm in diameter
	2	> 25 mm in diameter with < 50% surface area of the extremity segment involved (e.g., upper arm or thigh)
	3	≥ 50% surface area of the extremity segment involved (e.g., upper arm or thigh) or ulceration or secondary infection or phlebitis or sterile abscess or drainage
	4	Potentially life-threatening consequences (e.g., abscess, exfoliative dermatitis, necrosis involving dermis or deeper tissue)
Fever (as measured by axillary route)	1	> 37.5 to < 38.6°C
	2	≥ 38.6 to < 39.3°C
	3	≥ 39.3°C to < 40.0°C
	4	≥ 40.0°C
Drowsiness	1	Causes no or minimal interference with usual social & functional activities
	2	Causes greater than minimal interference with usual social & functional activities
	3	Causes inability to perform usual social & functional activities
	4	Potentially life-threatening with intervention indicated to prevent permanent impairment, persistent disability, or death
Irritability	1	Causes no or minimal interference with usual social & functional activities
	2	Causes greater than minimal interference with usual social & functional activities
	3	Causes inability to perform usual social & functional activities
	4	Potentially life-threatening with intervention indicated to prevent permanent impairment, persistent disability, or death
Anorexia	1	Loss of appetite without decreased oral intake
	2	Loss of appetite associated with decreased oral intake without significant weight loss
	3	Loss of appetite associated with significant weight loss
	4	Life-threatening consequences or aggressive intervention indicated (e.g., tube feeding, total parenteral nutrition)
Diarrhoea	1	Transient or intermittent episodes of unformed stools or increase of ≤ 3 stools over baseline per 24-hour period
	2	Persistent episodes of unformed to watery stools or increase of 4 to 6 stools over baseline per 24-hour period
	3	Increase of ≥ 7 stools per 24-hour period or IV fluid replacement indicated
	4	Life-threatening consequences (e.g., hypotensive shock)

Table: Grading scale to assess the severity of solicited adverse events for participants six years of age or older

Solicited AE	Grade	Definition
Pain at injection site	1	Pain or tenderness causing no or minimal limitation of use of limb
	2	Pain or tenderness causing greater than minimal limitation of use of limb
	3	Pain or tenderness causing inability to perform usual social & functional activities
	4	Pain or tenderness causing inability to perform basic self-care function or hospitalization indicated
Swelling/induration at injection site for subjects ≤ 15 years of age	1	≤ 25 mm in diameter
	2	> 25 mm in diameter with < 50% surface area of the extremity segment involved (e.g., upper arm or thigh)
	3	≥ 50% surface area of the extremity segment involved (e.g., upper arm or thigh) or ulceration or secondary infection or phlebitis or sterile abscess or drainage
	4	Potentially life-threatening consequences (e.g., abscess, exfoliative dermatitis, necrosis involving dermis or deeper tissue)
Swelling/induration at injection site for subjects > 15 years of age	1	< 50 mm in diameter or 6.25 to < 25 cm^2 surface area and symptoms causing no or minimal interference with usual social & functional activities
	2	≥ 50 to < 100 mm in diameter or ≥ 25 to < 100 cm^2 surface area or symptoms causing greater than minimal interference with usual social & functional activities
	3	≥ 100 mm in diameter or ≥ 100 cm^2 surface area or ulceration or secondary infection or phlebitis or sterile abscess or drainage or symptoms causing inability to perform usual social & functional activities
	4	Potentially life-threatening consequences (e.g., abscess, exfoliative dermatitis, necrosis involving dermis or deeper tissue)
Fever (as measured by axillary route)	1	> 37.5 to < 38.6°C
	2	≥ 38.6 to < 39.3°C
	3	≥ 39.3°C to < 40.0°C
	4	≥ 40.0°C
Headache	1	Causes no or minimal interference with usual social & functional activities
	2	Causes greater than minimal interference with usual social & functional activities
	3	Causes inability to perform usual social & functional activities
	4	Causes inability to perform basic self-care functions or hospitalization indicated or headache with significant impairment of alertness or other neurologic function
Fatigue	1	Causes no or minimal interference with usual social & functional activities
	2	Causes greater than minimal interference with usual social & functional activities
	3	Causes inability to perform usual social & functional activities
	4	Incapacitating symptoms of fatigue causing inability to perform basic self-care functions
Myalgia (Muscle Pain)	1	Causes no or minimal interference with usual social & functional activities
	2	Causes greater than minimal interference with usual social & functional activities
	3	Causes inability to perform usual social & functional activities
	4	Disabling muscle pain causing inability to perform basic self-care functions
Arthralgia (Joint Pain)	1	Causes no or minimal interference with usual social & functional activities
	2	Causes greater than minimal interference with usual social & functional activities
	3	Causes inability to perform usual social & functional activities
	4	Disabling joint pain causing inability to perform basic self-care functions

Solicited AE	Grade	Definition
Anorexia	1	Loss of appetite without decreased oral intake
	2	Loss of appetite associated with decreased oral intake without significant weight loss
	3	Loss of appetite associated with significant weight loss
	4	Life-threatening consequences or aggressive intervention indicated (e.g., tube feeding, total parenteral nutrition)
Diarrhoea	1	Transient or intermittent episodes of unformed stools OR increase of ≤ 3 stools over baseline per 24-hour period
	2	Persistent episodes of unformed to watery stools OR increase of 4 to 6 stools over baseline per 24-hour period
	3	Increase of ≥ 7 stools per 24-hour period OR IV fluid replacement indicated
	4	Life-threatening consequences (e.g., hypotensive shock)

Unsolicited adverse events

The severity of all unsolicited adverse events listed specifically as an event in the Division of AIDS (DAIDS) Table for Grading the Severity of Adult and Pediatric Adverse Events, corrected version 2.1, July 2017, of the US National Institutes of Health, were assessed based on this Table.

The following grading scale was used to grade the severity of all unsolicited AEs that are not listed as a specific event in the DAIDS Table cited:

- Grade 1 = Causes no or minimal interference with usual social & functional activities
- Grade 2 = Causes greater than minimal interference with usual social & functional activities
- Grade 3 = Causes inability to perform usual social & functional activities
- Grade 4 = Potentially life-threatening symptoms causing inability to perform basic self-care functions with intervention indicated to prevent permanent impairment, persistent disability, or death

Statistical analysis

All statistical analyses were performed using SAS® software version 9.4 (SAS Institute).

Sample size calculation

Immunogenicity

To demonstrate comparability of immune responses, a Prospective Alpha Allocation Scheme (PAAS) will be used for multiplicity adjustment. A significance level of 0.02 will be applied to the non-inferiority testing for seroresponse rate and a significance level of 0.0051 will be applied to the non-inferiority testing for GMTs.

The power to show comparability of immune responses in terms of percentage of subjects with an rSBA seroresponse against serogroups A, C, W, Y, and X 28 days following immunization, based on various assumed underlying percentages for seroresponse in reference of the data from the phase 2 trial conducted in Mali and the sample size of 400 in each NmCV-5 age group and 200 in each Men ACWY-D age group, is calculated using a one-sided Miettinen & Nurminen score test and displayed in the table below. Additional assumptions include a one-sided significance level of 0.02 and a non-inferiority margin of minus 10% for all five serogroups, and a 5% dropout rate:

Power to show comparability of immune responses in terms of percentage of subjects with an rSBA seroresponse against serogroups A, C, W, Y, and X 28 days following immunization:

**Serogroup**	**Subjects with seroresponse in NmCV-5 group**	**Subjects with seroresponse in Men ACWY-D group**	**Power**
A	90%	90%	97%
C	75%	70%	96%
W	90%	90%	97%
Y	90%	85%	> 99%
X*	75%	70%	96%
**Overall**			87%

* For serogroup X, seroresponse rate in the NmCV-5 group will be compared with the lowest seroresponse rate among serogroups A, C, W, and Y in the Men ACWY-D group.

The power to show comparability of immune responses in terms of GMTs against serogroups A, C, W, Y, and X 28 days following immunization, based on various assumed standard deviations (SD) of log_2-transformed rSBA titres and the sample size of 400 in NmCV-5 group and 200 in Men ACWY-D group,

was calculated using a one-sided two-sample t-test and is displayed in the table below. The SDs were estimated from the phase 2 trial conducted in Mali. Additional assumptions include a one-sided significance level of 0.0051, a difference in means of $\log_2$-transformed rSBA titres between NmCV-5 and Men ACWY-D groups of 0.17, a non-inferiority margin of 0.5 for all five serogroups, and a 5% dropout rate:

Power to show comparability of immune responses in terms of GMTs against serogroups A, C, W, Y, and X 28 days following immunization:

**Serogroup**	**Assumed SD of $\log_2$ rSBA titres in the NmCV-5 group**	**Assumed SD of $\log_2$ rSBA titres in the Men ACWY-D group**	**Power**
A	1.57	1.90	> 99%
C	1.82	3.35	97%
W	2.57	3.74	90%
Y	2.22	3.50	95%
X*	1.42	3.35	98%
**Overall**			**81%**

* For serogroup X, the GMT in the NmCV-5 group was to be compared with the lowest GMT among serogroups A, C, W, and Y in the Men ACWY-D group.

Safety

The study was designed to have a 95% probability to detect at least one given adverse event in subjects administered NmCV-5 (N = 400), if the true incidence of the adverse event was 0.8% and the subject discontinuation rate was 5%.

The minimum difference in percentage of subjects with any given adverse event between the NmCV-5 (N=400) and Men ACWY-D (N=200) groups that could be detected with 80% power is provided in the table below, based on a two-sided Fisher's Exact Test with a significance level of 0.05 and a 5% subject discontinuation rate:

Minimum difference in percentage of subjects with any given AE between the NmCV-5 and Men ACWY-D groups that could be detected with 80% power within each age group

Assumed percentage of subjects with any given AE in the Men ACWY-D group	Minimum difference in percentage of subjects with the given AE between the groups that could be detected with 80% power
1%	4.6%
3%	6.2%
5%	7.3%
10%	9.1%
15%	10.3%
20%	11.2%
25%	11.7%

Since the subjects were recruited as per age stratification with equal distribution of subjects in each of the three age groups, to facilitate further comparison per age group, the overall sample size was extrapolated to 1800, which took into account a dropout rate of 5%.

Analysis populations

The primary immunogenicity analysis was conducted in the per protocol population which included all participants who were randomized and vaccinated, who had serology results available, in the absence of protocol deviations considered likely to impact on the immunogenicity assessment. A supportive immunogenicity analysis was performed in a full analysis population which included all participants who were randomized and vaccinated, who had a serology result available irrespective of any protocol deviations. Participants in the full analysis population were analysed as randomized irrespective of the final vaccine received. The results of this supportive analysis aligned with those of the analysis conducted in the per protocol population (data not shown).

The safety analysis was conducted in a safety population which included all vaccinated participants who provided any safety data.

Representativeness of Study Participants

**Disease under investigation**	Meningitis and septicaemia caused by *Neisseria meningitidis* serogroups A, C, W, Y and X
**Sex**	The disease affects both sexes, any sex imbalances (male > female) are marginal
**Age**	All age groups may be affected although adults are a key age group impacted by epidemic meningitis in the African meningitis belt and hence are the target group for mass vaccination campaigns.
**Race and ethnic groups**	Although meningitis and septicaemia due to *Neisseria meningitidis* serogroups A, C, W, Y and X can occur in any race or ethnic group the NmCV-5 vaccine being assessed in this trial aims to prevent epidemic meningitis in the African meningitis belt
**Geography**	Although meningitis and septicaemia due to *Neisseria meningitidis* serogroups A, C, W, Y and X occur worldwide the highest incidence of disease occurs in the African meningitis belt which stretches from The Gambia and Senegal in west Africa to Ethiopia in east Africa
**Overall representativeness of this trial**	Both The Gambia and Mali are in the African meningitis belt. Both countries have experienced epidemics of meningococcal meningitis necessitating mass vaccination campaigns. All participants recruited to the trial were of African origin and resident in these two countries. The ethnic breakdown of the participants enrolled was representative of the populations in these two countries and approximately 50% of participant were female. The trial independently assessed the safety and immunogenicity of NmCV-5 in three age groups (2–10-year-olds, 11-17-year-olds, 18-29-year-olds). Thus, the trial data support the use of NmCV-5 across this full age range. Participants who were pregnant were not enrolled so the trial should not be used to support vaccination of pregnant females. Other eligibility criteria aimed to ensure the robustness of the safety and immunogenicity data generated. There is no reason to expect the safety profile of the vaccine to be different in those excluded for reasons other than pregnancy although the immunogenicity of the vaccine may be modified.<br><br>The participants recruited were thus highly representative of the future target population of NmCV-5 in mass vaccination campaigns in the African meningitis belt. However, it is also likely to be reasonable to extrapolate the findings to other populations of a comparable age.

Supplementary figures

Figure S1: Screening, enrolment, and follow-up of participants

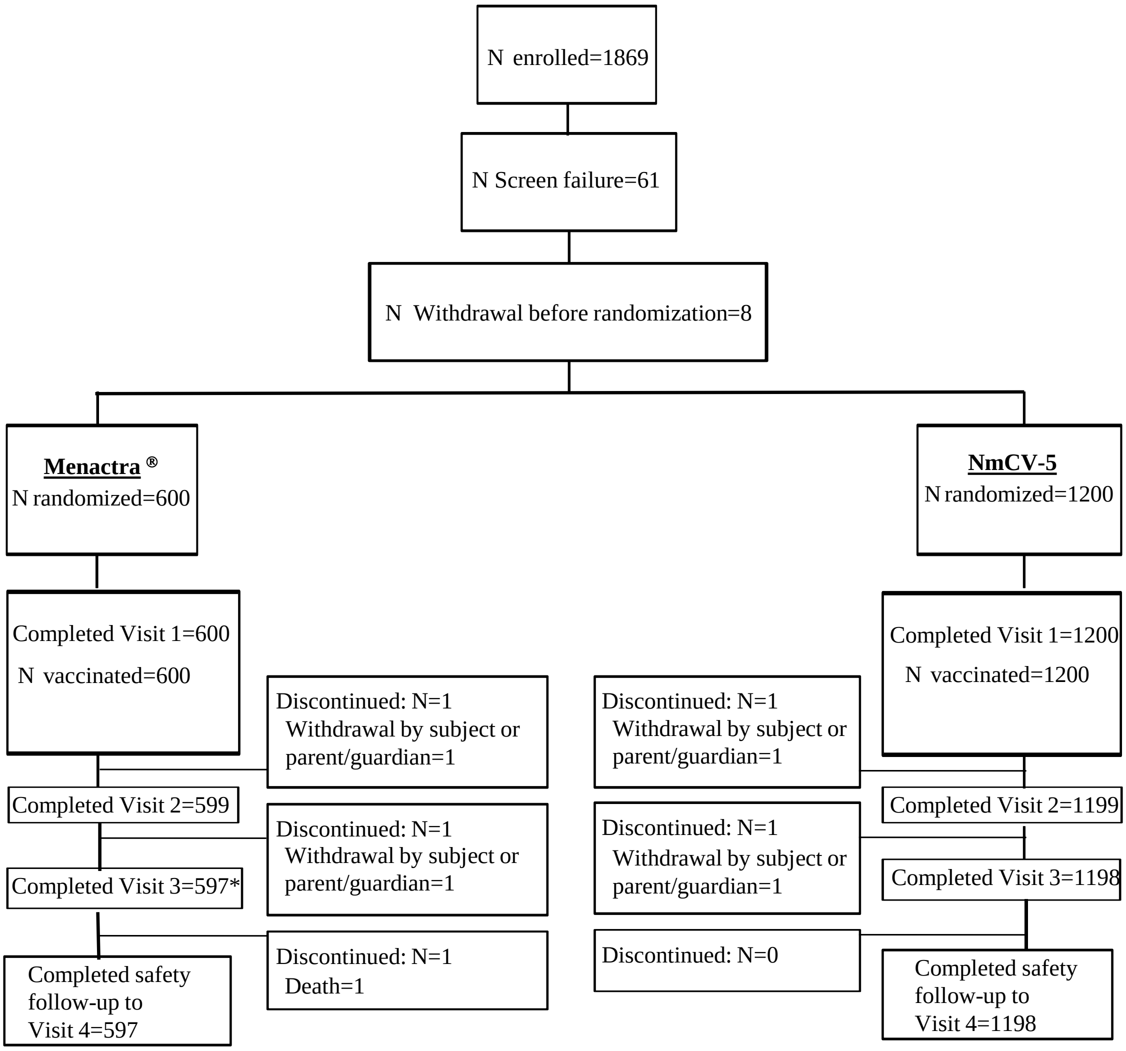

*One participant in the 11-17 years of age group completed Visit 2 and Visit 4 safety assessment but did not attend Visit 3.

Supplementary tables

Immunogenicity

Table S1: Percentage of participants with an rSBA titre >= 8 and >= 128 on the day of vaccination and on day 28 post-vaccination, per protocol population

	2 to 10 years				11 to 17 years				18 to 29 years				Overall			
	Day 0		Day 28		Day 0		Day 28		Day 0		Day 28		Day 0		Day 28	
	NmCV-5 n/N % (95% CI)	MenACWY-D n/N % (95% CI)	NmCV-5 n/N % (95% CI)	MenACWY-D n/N % (95% CI)	NmCV-5 n/N % (95% CI)	MenACWY-D n/N % (95% CI)	NmCV-5 n/N % (95% CI)	MenACWY-D n/N % (95% CI)	NmCV-5 n/N % (95% CI)	MenACWY-D n/N % (95% CI)	NmCV-5 n/N % (95% CI)	MenACWY-D n/N % (95% CI)	NmCV-5 n/N % (95% CI)	MenACWY-D n/N % (95% CI)	NmCV-5 n/N % (95% CI)	MenACWY-D n/N % (95% CI)
**rSBA titre $\geq$ 8**																
**A**	385/389	194/195	388/388	199/199	393/393	197/197	389/389	192/192	397/397	192/192	395/395	192/192	1175/1179	583/584	1172/1172	583/583
	99.0% (97.4-99.7)	99.5% (97.2-100)	100% (99.1-100)	100% (98.2-100)	100% (99.1-100)	100% (98.1-100)	100% (99.1-100)	100% (98.1-100)	100% (99.1-100)	100% (98.1-100)	100% (99.1-100)	100% (98.1-100)	99.7% (99.1-99.9)	99.8% (99.0-100)	100% (99.7-100)	100% (99.4-100)
**C**	67/388	23/192	395/396	196/197	129/374	64/183	395/396	197/198	186/379	89/188	396/398	193/193	382/1141	176/563	1186/1190	586/588
	17.3% (13.6-21.4)	12.0% (7.7-17.4)	99.7% (98.6-100)	99.5% (97.2-100)	34.5% (29.7-39.6)	35.0% (28.1-42.4)	99.7% (98.6-100)	99.5% (97.2-100)	49.1% (43.9-54.2)	47.3% (40.0-54.7)	99.5% (98.2-99.9)	100% (98.1-100)	33.5% (30.7-36.3)	31.3% (27.5-35.3)	99.7% (99.1-99.9)	99.7% (98.8-100)
**W**	158/373	77/179	395/396	199/199	200/360	112/176	394/395	195/197	273/376	133/184	393/394	192/193	631/1109	322/539	1182/1185	586/589
	42.4% (37.3-47.6)	43.0% (35.7-50.6)	99.7% (98.6-100)	100% (98.2-100)	55.6% (50.3-60.8)	63.6% (56.1-70.7)	99.7% (98.6-100)	99.0% (96.4-99.9)	72.6% (67.8-77.1)	72.3% (65.2-78.6)	99.7% (98.6-100)	99.5% (97.1-100)	56.9% (53.9-59.8)	59.7% (55.5-63.9)	99.7% (99.3-99.9)	99.5% (98.5-99.9)
**Y**	306/356	163/188	398/398	198/198	309/354	154/174	397/397	198/199	296/351	154/179	391/391	194/194	911/1061	471/541	1186/1186	590/591
	86.0% (81.9-89.4)	86.7% (81.0-91.2)	100% (99.1-100)	100% (98.2-100)	87.3% (83.4-90.6)	88.5% (82.8-92.8)	100% (99.1-100)	99.5% (97.2-100)	84.3% (80.1-88.0)	86.0% (80.1-90.8)	100% (99.1-100)	100% (98.1-100)	85.9% (83.6-87.9)	87.1% (83.9-89.8)	100% (99.7-100)	99.8% (99.1-100)
**X**	369/386	187/195	399/399	184/187	348/377	180/193	392/392	176/180	329/378	159/180	396/396	146/156	1046/1141	526/568	1187/1187	506/523
	95.6% (93.0-97.4)	95.9% (92.1-98.2)	100% (99.1-100)	98.4% (95.4-99.7)	92.3% (89.1-94.8)	93.3% (88.8-96.4)	100% (99.1-100)	97.8% (94.4-99.4)	87.0% (83.2-90.3)	88.3% (82.7-92.6)	100% (99.1-100)	93.6% (88.5-96.9)	91.7% (89.9-93.2)	92.6% (90.1-94.6)	100% (99.7-100)	96.7% (94.8-98.1)
**rSBA titre $\geq$ 128**																
**A**	385/389	194/195	388/388	199/199	389/393	197/197	389/389	192/192	393/397	188/192	395/395	192/192	1167/1179	579/584	1172/1172	583/583
	99.0% (97.4-99.7)	99.5% (97.2-100)	100% (99.1-100)	100% (98.2-100)	99.0% (97.4-99.7)	100% (98.1-100)	100% (99.1-100)	100% (98.1-100)	99.0% (97.4-99.7)	97.9% (94.8-99.4)	100% (99.1-100)	100% (98.1-100)	99.0% (98.2-99.5)	99.1% (98.0-99.7)	100% (99.7-100)	100% (99.4-100)
**C**	37/388	10/192	389/396	181/197	76/374	34/183	392/396	190/198	108/397	53/188	393/398	185/193	221/1141	97/563	1174/1190	556/588
	9.5% (6.8-12.9)	5.2% (2.5-9.4)	98.2% (96.4-99.3)	91.9% (87.1-95.3)	20.3% (16.4-24.8)	18.6% (13.2-25.0)	99.0% (97.4-99.7)	96.0% (92.2-98.2)	28.5% (24.0-33.3)	28.2% (21.9-35.2)	98.7% (97.1-99.6)	95.9% (92.0-98.2)	19.4% (17.1-21.8)	17.2% (14.2-20.6)	98.7% (97.8-99.2)	94.6% (92.4-96.2)
**W**	141/373	62/179	395/396	199/199	177/360	102/176	394/395	195/197	238/376	115/184	393/394	192/193	556/1109	279/539	1182/1185	586/589
	37.8% (32.9-42.9)	34.6% (27.7-42.1)	99.7% (98.6-100)	100% (98.2-100)	49.2% (43.9-54.5)	58.0% (50.3-65.3)	99.7% (98.6-100)	99.0% (96.4-99.9)	63.3% (58.2-68.2)	62.5% (55.1-69.5)	99.7% (98.6-100)	99.5% (97.1-100)	50.1% (47.2-53.1)	51.8% (47.5-56.1)	99.7% (99.3-99.9)	99.5% (98.5-99.9)
**Y**	296/356	155/188	398/398	198/198	299/354	152/174	397/397	198/199	279/351	147/179	391/391	194/194	874/1061	454/541	1186/1186	590/591
	83.1% (78.8-86.9)	82.4% (76.2-87.6)	100% (99.1-100)	100% (98.2-100)	84.5% (80.3-88.1)	87.4% (81.5-91.9)	100% (99.1-100)	99.5% (97.2-100)	79.5% (74.9-83.6)	82.1% (75.7-87.4)	100% (99.1-100)	100% (98.1-100)	82.4% (79.9-84.6)	83.9% (80.5-86.9)	100% (99.7-100)	99.8% (99.1-100)
**X**	365/386	182/195	399/399	180/187	340/377	178/193	392/392	171/180	310/378	150/180	395/396	131/156	1015/1141	510/568	1186/1187	482/523
	94.6% (91.8-96.6)	93.3% (88.9-96.4)	100% (99.1-100)	96.3% (92.4-98.5)	90.2% (86.7-93.0)	92.2% (87.5-95.6)	100% (99.1-100)	95.0% (90.7-97.7)	82.0% (77.8-85.8)	83.3% (77.1-88.5)	99.7% (98.6-100)	84.0% (77.3-89.4)	89.0% (87.0-90.7)	89.8% (87.0-92.2)	99.9% (99.5-100)	92.2% (89.5-94.3)

rSBA – rabbit complement serum bactericidal activity; Nm-CV-5 – Serum Institute of India Pvt. Ltd pentavalent ACWYX meningococcal conjugate vaccine; MenACWY-D – Sanofi Pasteur quadrivalent meningococcal conjugate vaccine (Menactra®); n/N – number of participants with a rSBA titres $\geq$ 8 or 128/number of evaluable participants; CI – confidence interval.

Table S2: Baseline and post-vaccination serogroup-specific rSBA GMT and geometric mean fold rise, per protocol population

	**2−10 years**				**11−17 years**				**18−29 years**				**Overall**			
	**Day 1**		**Day 29**		**Day 1**		**Day 29**		**Day 1**		**Day 29**		**Day 1**		**Day 29**	
**Serogroup**	**NmCV-5** n GMT (95% CI)	**MenACWY-D** n GMT (95% CI)	**NmCV-5** n GMT (95% CI) **GMFR** (95% CI)	**MenACWY-D** n GMT (95% CI) **GMFR** (95% CI)	**NmCV-5** n GMT (95% CI)	**MenACWY-D** n GMT (95% CI)	**NmCV-5** n GMT (95% CI) **GMFR** (95% CI)	**MenACWY-D** n GMT (95% CI) **GMFR** (95% CI)	**NmCV-5** n GMT (95% CI)	**MenACWY-D** n GMT (95% CI)	**NmCV-5** n GMT (95% CI) **GMFR** (95% CI)	**MenACWY-D** n GMT (95% CI) **GMFR** (95% CI)	**NmCV-5** n GMT (95% CI)	**MenACWY-D** n GMT (95% CI)	**NmCV-5** n GMT (95% CI) **GMFR** (95% CI)	**MenACWY-D** n GMT (95% CI) **GMFR** (95% CI)
A	389	195	388	199	393	197	389	192	397	192	395	192	1179	584	1172	583
	1418.8	1466.3	9250.1	5682.7	1605.6	1772.9	9463.9	4871.0	1289.4	1235.5	5900.3	3796.9	1431.6	1477.7	8009.9	4729.7
	(1258.0–1600.1)	(1262.6–1702.8)	(8529.3–10,031.8)	(5079.4–6357.6)	(1465.3–1759.3)	(1574.0–1996.9)	(8762.4–10221.7)	(4349.1–5455.6)	(1180.4–1408.4)	(1070.7–1425.6)	(5419.2–6424.2)	(3363.2–4286.7)	(1350.6–1517.5)	(1364.2–1600.6)	(7631.7–8407.0)	(4420.0–5061.2)
			6.6 (5.7–7.6)	3.8 (3.3–4.5)			5.8 (5.2–6.5)	2.8 (2.4–3.2)			4.6 (4.1–5.1)	3.1 (2.7–3.6)			5.6 (5.2–6.0)	3.2 (3.0–3.5)
C	388	192	396	197	374	183	396	198	379	188	398	193	1141	563	1190	588
	4.0	3.2	3084.7	1020.4	8.6	8.6	8021.7	2906.5	15.1	14.7	7040.2	2153.6	8.0	7.4	5587.2	1854.9
	(3.4–4.7)	(2.7–3.9)	(2684.4–3544.7)	(814.6–1278.3)	(6.9–10.7)	(6.3–11.8)	(6973.0–9228.2)	(2322.2–3637.8)	(12.0–19.1)	(10.5–20.5)	(6036.8–8210.5)	(1703.7–2722.4)	(7.1–9.0)	(6.2–8.7)	(5123.7–6092.5)	(1619.6–2124.4)
			754.0 (620.7–916.0)	316.7 (240.8–416.4)			929.2 (730.8–1181.5)	330.4 (232.8–469.0)			461.9 (351.4–607.2)	150.9 (105.0–217.0)			685.9 (597.6–787.3)	250.9 (207.1–304.1)
W	373	179	396	199	360	176	395	197	376	184	394	193	1109	539	1185	589
	18.6	16.5	28,888.0	11,208.2	36.8	61.3	30,280.0	13,453.9	95.0	93.3	27,773.5	12336.7	40.4	45.8	28,963.4	12,294.6
	(14.2–24.4)	(11.4–23.9)	(25,399.7–32,855.3)	(8887.3–14,135.2)	(27.7–49.0)	(41.0–91.7)	(26,538.4–34,549.0)	(10,845.1–16,690.2)	(72.3–124.7)	(63.0–138.1)	(24,082.4–32,030.4)	(9713.9–15,667.8)	(34.2–47.5)	(36.3–57.7)	(26,804.6–31,295.9)	(10,778.9–14,023.4)
			1559.1 (1190.7–2041.4)	658.5 (456.3–950.4)			833.0 (623.1–1113.8)	221.8 (145.6–337.8			308.2 (229.6–413.6)	132.0 (89.7–194.3)			736.3 (622.0–871.6)	267.9 (212.5–337.8)
Y	356	188	398	198	354	174	397	199	351	179	391	194	1061	541	1186	591
	194.2	185.8	10,768.1	4362.4	227.2	266.4	11,256.2	5154.6	193.4	204.5	10518.3	4967.7	204.3	215.3	10,844.8	4815.6
	(156.9–240.3)	(140.1–246.3)	(9831.2–11,794.3)	(3701.0–5142.0)	(184.5–279.7)	(198.6–357.4)	(10,286.9–12,317.0)	(4359.4–6095.0)	(153.7–243.3)	(150.8–277.3)	(9450.9–11,706.2)	(4227.8–5837.0)	(180.3–231.5)	(181.9–254.9)	(10,260.2–11,462.8)	(4380.9–5293.4)
			56.2 (45.2–69.7)	24.1 (18.5–31.5)			49.7 (40.0–61.9)	20.6 (15.1–28.3)			55.5 (43.6–70.6)	25.8 (19.3–34.4)			53.7 (47.2–61.1)	23.4 (19.9–27.7)
X	386	195	399	187	377	193	392	180	378	180	396	156	1141	568	1187	523
	701.0	715.1	39,737.0	1031.6	501.7	604.0	44,572.8	835.0	286.3	257.0	17,327.5	426.7	466.6	488.2	31,290.4	737.1
	(595.6–825.2)	(566.0–903.5)	(35,838.0–44,060.2)	(845.1–1259.3)	(415.5–605.9)	(469.8–776.5)	(39,929.6–49,756.0)	(682.6–1021.4)	(230.3–356.0)	(189.8–347.9)	(15,369.3–19,535.2)	(311.7–584.2)	(417.2–521.8)	(418.2–570.0)	(29,222.2–33,505.1)	(641.3–847.4)
			57.3 (47.6–68.9)	1.2 (1.1–1.4)			87.9 (70.9–109.1)	1.1 (1.0–1.3)			63.1 (49.8–79.9)	1.2 (1.0–1.4)			68.0 (60.2–77.0)	1.2 (1.1–1.3)

rSBA – rabbit complement serum bactericidal activity; Nm-CV-5 – Serum Institute of India Pvt. Ltd pentavalent ACWYX meningococcal conjugate vaccine; MenACWY-D – Sanofi Pasteur quadrivalent meningococcal conjugate vaccine (Menactra®); n – number of participants included in the analysis; CI – confidence interval; GMT – geometric mean titres; GMFR – geometric mean fold rise; GMT of antibodies measured by rSBA against each of the five serogroups and 95% CI were calculated by exponentiating the

corresponding log_2-transformed mean and its two-sided 95% CI limits. The log_2-transformed rSBA titres were used to construct a two-sided 95% CI for the mean difference from Visit 1 to Visit 3 for each treatment using paired t-test. The mean difference and corresponding 95% CI limits were exponentiated to obtain the GMT ratio and the corresponding 95% CI; rSBA titre values below lower limit of quantification (LLOQ) are set to LLOQ/2 for analysis.

Safety

Table S3: Unsolicited adverse events by MedDRA[1] preferred term recorded in at least five participants - safety population

	2−10 years		11−17 years		18−29 years		Overall		
**Preferred Term**	**NmCV-5** N=400 n (%) [e]	**MenACWY-D** N=200 n (%) [e]	**NmCV-5** N=400 n (%) [e]	**MenACWY-D** N=200 n (%) [e]	**NmCV-5** N=400 n (%) [e]	**MenACWY-D** N=200 n (%) [e]	**NmCV-5** N=1200 n (%) [e]	**MenACWY-D** N=600 n (%) [e]	**Total** N=1800 n (%) [e]
Upper Respiratory Tract Infection	33 (8.3) [35]	16 (8.0) [19]	9 (2.3) [10]	6 (3.0) [6]	14 (3.5) [14]	5 (2.5) [5]	56 (4.7) [59]	27 (4.5) [30]	83 (4.6) [89]
Malaria	3 (0.8) [3]	0 (0.0) [0]	3 (0.8) [3]	3 (1.5) [3]	8 (2.0) [8]	6 (3.0) [6]	14 (1.2) [14]	9 (1.5) [9]	23 (1.3) [23]
Pharyngitis	4 (1.0) [4]	3 (1.5) [3]	1 (0.3) [1]	1 (0.5) [1]	3 (0.8) [3]	3 (1.5) [3]	8 (0.7) [8]	7 (1.2) [7]	15 (0.8) [15]
Bronchitis	5 (1.3) [5]	5 (2.5) [5]	1 (0.3) [1]	0 (0.0) [0]	1 (0.3) [1]	0 (0.0) [0]	7 (0.6) [7]	5 (0.8) [5]	12 (0.7) [12]
Dyspepsia	0 (0.0) [0]	0 (0.0) [0]	3 (0.8) [3]	2 (1.0) [2]	6 (1.5) [6]	1 (0.5) [1]	9 (0.8) [9]	3 (0.5) [3]	12 (0.7) [12]
Gastroenteritis	0 (0.0) [0]	2 (1.0) [2]	5 (1.3) [5]	1 (0.5) [1]	2 (0.5) [2]	0 (0.0) [0]	7 (0.6) [7]	3 (0.5) [3]	10 (0.6) [10]
Lower Respiratory Tract Infection	1 (0.3) [1]	1 (0.5) [1]	0 (0.0) [0]	0 (0.0) [0]	4 (1.0) [4]	1 (0.5) [1]	5 (0.4) [5]	2 (0.3) [2]	7 (0.4) [7]
Abdominal Pain	2 (0.5) [2]	0 (0.0) [0]	2 (0.5) [2]	0 (0.0) [0]	1 (0.3) [1]	2 (1.0) [2]	5 (0.4) [5]	2 (0.3) [2]	7 (0.4) [7]
Headache	0 (0.0) [0]	0 (0.0) [0]	2 (0.5) [2]	2 (1.0) [2]	2 (0.5) [2]	1 (0.5) [1]	4 (0.3) [4]	3 (0.5) [3]	7 (0.4) [7]
Helminthic Infection	5 (1.3) [5]	0 (0.0) [0]	0 (0.0) [0]	0 (0.0) [0]	1 (0.3) [1]	0 (0.0) [0]	6 (0.5) [6]	0 (0.0) [0]	6 (0.3) [6]
Conjunctivitis	0 (0.0) [0]	1 (0.5) [1]	2 (0.5) [2]	0 (0.0) [0]	1 (0.3) [1]	2 (1.0) [2]	3 (0.3) [3]	3 (0.5) [3]	6 (0.3) [6]
Diarrhoea	3 (0.8) [3]	0 (0.0) [0]	0 (0.0) [0]	1 (0.5) [1]	1 (0.3) [1]	1 (0.5) [1]	4 (0.3) [4]	2 (0.3) [2]	6 (0.3) [6]
Bacterial conjunctivitis	3 (0.8) [3]	1 (0.5) [1]	1 (0.3) [1]	0 (0.0) [0]	0 (0.0) [0]	0 (0.0) [0]	4 (0.3) [4]	1 (0.2) [1]	5 (0.3) [5]
Nasopharyngitis	3 (0.8) [3]	1 (0.5) [1]	0 (0.0) [0]	1 (0.5) [1]	0 (0.0) [0]	0 (0.0) [0]	3 (0.3) [3]	2 (0.3) [2]	5 (0.3) [5]
Tension headache	0 (0.0) [0]	0 (0.0) [0]	3 (0.8) [3]	1 (0.5) [1]	0 (0.0) [0]	1 (0.5) [1]	3 (0.3) [3]	2 (0.3) [2]	5 (0.3) [5]
Back Pain	0 (0.0) [0]	0 (0.0) [0]	1 (0.3) [1]	0 (0.0) [0]	2 (0.5) [2]	2 (1.0) [2]	3 (0.3) [3]	2 (0.3) [2]	5 (0.3) [5]

Nm-CV-5 – Serum Institute of India Pvt. Ltd pentavalent ACWYX meningococcal conjugate vaccine; MenACWY-D – Sanofi Pasteur quadrivalent meningococcal conjugate vaccine (Menactra®); N = overall number of participants included in the safety population; n – number of participants experiencing at least one of a given event; % proportion of the participants experiencing a given event (n/N); [e] – number of individual events recorded; [1] MedDRA, version 22.0.
